# The Nonlinear and Distinct Responses of Ocean Heat Content and Anthropogenic Carbon to Ice Sheet Freshwater Discharge in a Warming Climate

**DOI:** 10.1029/2024EF004475

**Published:** 2024-11-24

**Authors:** Tessa Gorte, Nicole S. Lovenduski, Cara Nissen, Jan T. M. Lenaerts, Jeffrey B. Weiss

**Affiliations:** ^1^ Department of Atmospheric and Oceanic Sciences University of Colorado Boulder Boulder CO USA; ^2^ Institute of Arctic and Alpine Research University of Colorado Boulder Boulder CO USA

**Keywords:** Antarctica, Greenland, ocean heat content, ocean carbon content, climate model, linearity

## Abstract

Anthropogenic climate change will drive extensive mass loss across both the Antarctic (AIS) and Greenland Ice Sheets (GrIS), with the potential for global climate system feedbacks, especially in polar regions. Historically, the high‐latitude North Atlantic and Southern Ocean have been critical regions for anthropogenic heat and carbon uptake, but our understanding of how this uptake will be altered by future freshwater discharge is incomplete. We assess each ice sheet's impact on global ocean anthropogenic heat and carbon storage for a high‐emission scenario over the $21^{\text{st}}$‐century using a coupled Earth system model. We explore the impact of contemporaneous mass loss from both ice sheets on anthropogenic heat and carbon storage and quantify their linear and nonlinear contributions. Notably, added freshwater reduces ocean heat and carbon storage by 2,100, and the sum of individual freshwater effects differ from those induced by simultaneous freshwater discharge from both ice sheets. Combined AIS and GrIS freshwater engenders distinct anthropogenic storage anomalies—particularly in the high‐latitude Southern Ocean and North Atlantic. From 2080 to 2100, GrIS freshwater exerts primary control on the temporal evolution of global ocean heat storage, while global ocean carbon storage is modulated by the linear AIS and GrIS freshwater impacts. Nonlinear impacts of simultaneous ice sheet discharge have a non‐negligible contribution to the evolution of global ocean heat storage. Further, anthropogenic heat changes are realized more quickly in response to ice sheet discharge than anthropogenic carbon. Our results highlight the need to incorporate both ice sheets actively in climate models to accurately project future global climate.

## Key Points

- We disentangle the linear and nonlinear effects of Greenland and Antarctic Ice Sheet melt on ocean heat content and anthropogenic carbon
- Future anthropogenic carbon storage and ocean heat content have disparate responses to separate and combined ice sheet melt in polar regions
- Greenland freshwater is more influential than Antarctic freshwater in driving future changes in anthropogenic carbon and ocean heat content

## 1 Introduction

The global ocean has taken up roughly a third of all anthropogenic emissions of ${\text{CO}}_{2}$ ($C_{\text{ANTH}}$) over the course of the industrial period (DeVries et al., 2023; Friedlingstein et al., 2022; Gruber et al., 2023; Khatiwala et al., 2013) and over 90% of the excess heat over the last 50 years (Bindoff et al., 2007), thereby buffering the effects of climate change. The storage of excess heat and carbon is heavily dependent upon the physical and chemical state of the upper ocean including temperature, salinity, stratification, and carbonate chemistry (Gruber et al., 2023; Maier‐Reimer & Hasselmann, 1987; Sarmiento et al., 1992). Cooler, more saline surface waters destabilize the water column, promoting surface‐to‐depth transport of $C_{\text{ANTH}}$ and excess heat and thereby facilitating more heat and carbon uptake at the surface (Terhaar et al., 2021). Over half of all anthropogenic carbon stored in the global ocean is found in the upper 400 m, with the Southern Ocean (SO) south of 35°S alone accounting for over 40% of all $C_{\text{ANTH}}$ uptake (Gruber et al., 2019). Similarly, the SO south of 44°S dominates the global ocean uptake of heat (6.9 ZJ in heat uptake compared to 5.4 ZJ globally; Huguenin et al., 2022). The North Atlantic is also a critical region for the uptake of excess heat and $C_{\text{ANTH}}$ fluxes as the Atlantic Meridional Overturning Circulation (AMOC) drives surface‐to‐depth transport off the southern coast of the Greenland Ice Sheet (GrIS) (Gruber et al., 2002; Huguenin et al., 2022), but recent and projected trends in global heat uptake were shown to be dominated by the SO (Huguenin et al., 2022).

With increased carbon emissions and subsequent anthropogenic warming, the global ocean $C_{\text{ANTH}}$ inventory and the global excess ocean heat content (${\text{OHC}}_{\text{ANTH}}$) are projected to grow (Wanninkhof et al., 2013; Cheng et al., 2022; Terhaar et al., 2021; von Schuckmann et al., 2023), thereby shaping the trajectory of global climate change for the coming century and beyond (Abraham et al., 2013, 2022; Bronselaer & Zanna, 2020). Physical oceanographic changes will manifest first in the high latitudes (Bintanja & Oerlemans, 1995; Crook et al., 2011; Goosse et al., 2018; Holland & Bitz, 2003; Manabe & Stouffer, 1980)—including critical regions for heat and carbon uptake such as the Southern Ocean and the North Atlantic (Fletcher et al., 2006; Frölicher et al., 2015; Gruber et al., 2002; Huguenin et al., 2022; Khatiwala et al., 2013; Müller et al., 2023; Terhaar et al., 2021). Based on the $6^{th}$ Coupled Model Intercomparison Project (CMIP6) ensemble average under Shared Socioeconomic Pathway 5–8.5 (SSP5‐8.5), by the end of the $21^{\text{st}}$ century, the upper ocean is projected to take up an additional 25 ZJ (1 ZJ = $10^{21}$ J) of heat per year (Cheng et al., 2022) while anthropogenic carbon storage of the SO alone is projected to increase by ∼200 Pg C (1 Pg C = $10^{15}$g C) (Terhaar et al., 2021). At the same time, climate‐driven strengthening of upper ocean stratification will weaken overturning and, consequentially, the ability of the ocean to transfer the excess heat and anthropogenic carbon to greater depths (Davila et al., 2022; Swingedouw et al., 2007). Investigating their projected anthropogenic‐driven changes, Bronselaer and Zanna (2020) find a linear relationship between global anthropogenic heat and carbon changes over the $21^{\text{st}}$ century in the models assessed in their study. Although none of their studied models accounted for the expected increasing future freshwater discharge from ice sheets, these linked heat and carbon trends will act to further reduce the global ocean's ability to buffer climate‐change effects (Gruber et al., 2023).

One of the largest sources of projected oceanic change in the polar regions is meltwater from the Antarctic Ice Sheet (AIS) and the Greenland Ice Sheet (GrIS) which have been losing mass at rates of 107 Gt $y^{-1}$ and 261 Gt $y^{-1}$ (1 Gt = 1 Gigaton = $10^{12}$ kg), respectively, on average since 2002 (Velicogna et al., 2020). By 2100, the GrIS is expected to contribute 90 ± 50 cm to global mean sea level under Representative Concentration Pathway 8.5 (RCP8.5; Goelzer et al., 2020). The trend of the AIS contribution to global mean sea level is less well constrained, and end‐of‐century estimates range from −7.6 to 30.0 cm under the RCP8.5 scenario (Seroussi et al., 2020). Recent work demonstrated that ice sheet mass loss has significant ocean impacts, including surface cooling with subsurface warming, reduced deep convection and dense water formation, and, critically, strengthened upper ocean density gradients (Bronselaer & Zanna, 2020; Gorte et al., 2023; Li, England, et al., 2023; Menviel et al., 2015; Nissen et al., 2022; Park & Latif, 2019; Pauling et al., 2016; Sadai et al., 2020). Yet, most CMIP6 models do not have an ice sheet component or the capability for ice sheets to interact with the other model components (Nowicki et al., 2016; Swart et al., 2023). In lieu of active ice sheet modeling in global climate models (GCMs), there have been many efforts to account for ice sheet freshwater (FW) through FW sensitivity experiments—testing different magnitudes, timing, duration, and location of FW input (Bintanja et al., 2013; Bronselaer et al., 2018; Gorte et al., 2023; Park & Latif, 2019; Pauling et al., 2016; Purich & England, 2023; Sadai et al., 2020; Swart et al., 2023; Swart & Fyfe, 2013). Despite uncertainties arising from this one‐way, ice sheet‐to‐ocean FW coupling approach, these studies have demonstrated the robust changes to Southern Ocean physical properties when the ocean is subject to ice sheet FW input are worth further examination (Bintanja et al., 2013; Bronselaer et al., 2018; Gorte et al., 2023; Park & Latif, 2019; Pauling et al., 2016; Purich & England, 2023; Sadai et al., 2020; Swart & Fyfe, 2013).

Here, we use the Community Earth System Model version 2 (Danabasoglu et al., 2020) to quantify and diagnose the role of ice sheet FW discharge in the $21^{\text{st}}$ century evolution of global excess ocean heat content and anthropogenic carbon under the high‐emission scenario SSP5‐8.5. Our model sensitivity simulations are configured to separately assess the role of AIS and GrIS discharge, as well as the impact of their simultaneous melt. As we will demonstrate, excess ocean heat content and anthropogenic carbon respond differently to ice sheet discharge, and nonlinearity is pervasive in our results. Further, machine learning‐based analysis of our model output suggests that sea surface salinity (SSS) primarily drives changes in both quantities.

## 2 Methods

### 2.1 Model Simulations With CESM2

We perform four coupled climate simulations with the Community Earth System Model version 2 (CESM2; Danabasoglu et al., 2020), which differ in the representation of FW fluxes from the AIS and GrIS and will be described in more detail below: (a) a control simulation, (b) an AIS simulation, (c) a GrIS simulation, and (d) a combined AIS and GrIS simulation—hereafter referred to as the AGrIS simulation. The control and AIS simulations are identical to those used in Gorte et al. (2023). Each simulation is run with a ∼0.9×1.25° horizontal resolution in the ocean under historical CMIP6 greenhouse gas forcing from 1970 to 2014 and under SSP5‐8.5 greenhouse gas forcing from 2015 to 2100 (Meinshausen et al., 2020). The control simulation runs from 1970 to 2100 while the AIS, GrIS and AGrIS simulations branch off in 1992 and run until 2,100.

**Figure 1:**
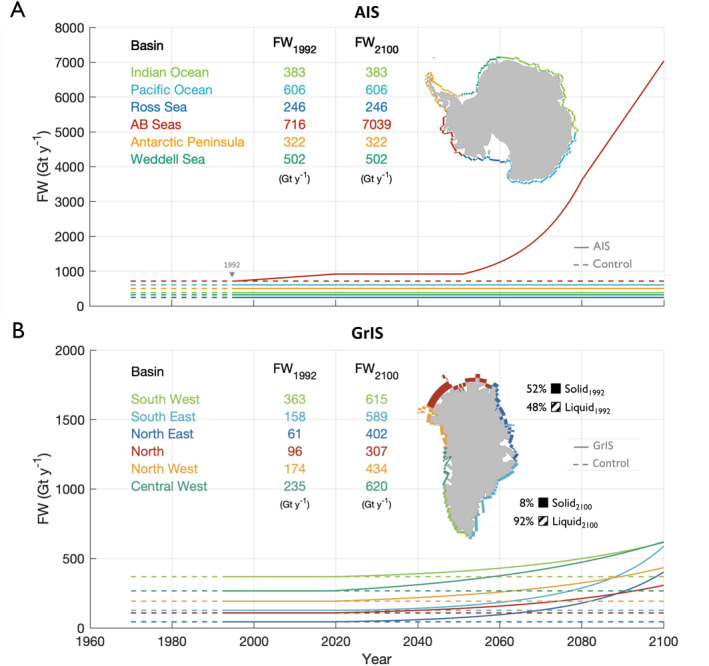
(a) Spatial distribution and temporal evolution of the total freshwater (FW) in Gt $y^{-1}$ flux for each Antarctic Ice Sheet (AIS) basin: the Indian Ocean (light green), the Pacific Ocean (light blue), the Ross Sea (dark blue), the Amundsen and Bellingshausen (AB) Seas (red), the Antarctic Peninsula (orange), and the Weddell Sea (dark green). The values displayed are the initial (1992) and final (2100) basin‐integrated FW fluxes in Gt $y^{-1}$. The time series show the temporal evolution of the basin‐integrated FW fluxes for the period (1970–2100) for the control (dashed) and AIS (solid) simulations. (b) Same as panel (a) but for the Greenland Ice Sheet (GrIS) and GrIS basins: South West (light green), South East (light blue), North East (dark blue), North (red), North West (orange), and Central West (dark green). Also displayed are the solid and liquid flux percentages for the integrated GrIS with the 1992 percentages shown to the upper right of the GrIS map and 2100 to the lower right in black. The solid‐to‐liquid FW flux ratios fluctuate with time for each GrIS basin. For more information on the solid‐to‐liquid FW flux ratio, see Figures S2–S3 and Table S2 in Supporting Information S1. (1 Gt = 1 Gigaton = $10^{12}$ kg).

In the control simulation, we do not allow for FW fluxes from either ice sheet to increase; instead, both ice sheets' FW contributions are held constant at their 1970 levels from 1970 to 2100 (dashed lines in Figure 1). To achieve this, we override the default mechanism for mass preservation for the AIS in CESM2, that is, the instantaneous transport of excess mass to the nearest coastal ocean grid cell as solid discharge when a 10 m of water equivalent mass threshold is exceeded. Instead, we point the model to prescribed solid and liquid flux values. The prescribed AIS FW discharge is the same for each month of the year: 231 Gt based on values reported by Lenaerts et al. (2015). Furthermore, we use findings reported in Lenaerts et al. (2015) to divide the AIS FW discharge across six ocean basins (Figure 1). The FW flux values for the GrIS are derived from historical, active‐Greenland CESM2 output which Noël et al. (2020) demonstrate yields realistic surface processes. In contrast to the AIS, the liquid FW discharge from the GrIS follows a strong seasonal cycle, peaking in July at 134 Gt and dropping to 0 Gt in the winter while the solid FW discharge is held constant over the annual cycle at 48 Gt. The GrIS FW discharge is also divided into six ocean basins based on Rignot et al. (2012). Annually, the combined solid and liquid discharge amounts to 2,775 Gt $y^{-1}$ from the AIS and 1088 Gt $y^{-1}$ from the GrIS in total FW fluxes (Table S1 in Supporting Information S1). The fluxes are modeled as salinity fluxes and are applied to the coastal surface grid cells. Each grid cell contributes a FW flux proportional to its area such that differently sized cells contribute the same total FW flux.

In the AIS simulation, GrIS FW fluxes match those of the control simulation while the AIS produces increasing FW fluxes from 1992 to 2100 (solid lines in Figure 1a). The AIS historical FW forcing is based on observational AIS mass balance data which were amalgamated by the Ice sheet Mass Balance Inter‐comparison Exercise (IMBIE) team (Shepherd et al., 2018) while the future FW forcing reflects recent results from ice sheet modeling (DeConto et al., 2021; Rignot et al., 2019). To produce the historical FW forcing, we apply a linear fit to the IMBIE team's AIS mass balance data such that the total AIS FW flux increases from 2775 Gt $y^{-1}$ in 1992 to ∼3160 Gt $y^{-1}$ by 2020 (Figure 1; Rignot et al., 2019). To generate the future AIS FW flux forcing, we follow results published by DeConto et al. (2021). Using a combination of GCMs and ice sheet models under RCP8.5 atmospheric conditions, DeConto et al. (2021) project a roughly constant contribution from the AIS to the global mean sea level through ∼2050 and a quasi‐exponentially increasing contribution thereafter. Projecting an AIS discharge rate of ∼6500 Gt $y^{-1}$ by 2100, DeConto et al. (2021) results are higher than the mean, but captured within the model spread (5000–8000 Gt $y^{-1}$) of the Ice Sheet Model Intercomparison Project for CMIP6 (ISMIP6; Nowicki et al., 2016). Following DeConto et al. (2021) projections, our total AIS FW forcing increases linearly from 2775 Gt $y^{-1}$ to ∼3160 Gt $y^{-1}$ from 1992 to 2020, remains at ∼3160 Gt $y^{-1}$ from 2021 to 2050, and then increases nonlinearly from ∼3160 Gt $y^{-1}$ to 9098 Gt $y^{-1}$ from 2051 to 2100 (Figure 1, Table S1 in Supporting Information S1). The IS‐wide mass balance data from Rignot et al. (2019) indicate that much of the historical AIS mass loss is concentrated in the West Antarctic Ice Sheet (WAIS) region along the coasts of the Amundsen and Bellingshausen Seas (AB Seas; 95°W to 145°W; Figure S1 in Supporting Information S1). Therefore, we distribute the area‐weighted, excess AIS FW evenly across the coastal ocean grid cells in the AB Seas ocean basin. With limited information to suggest the AIS experiences significant seasonality in solid or liquid discharge, distributing the liquid and solid fluxes (salinity fluxes in CESM2 with no temperature or momentum) to the surface coastal grid cells means the solid and liquid freshwater terms can be combined and treated as one, total freshwater flux for each AIS basin (Figure 1). This is a potential caveat of this work as we are constrained by limited data from observations and models to indicate the heterogeneity of future AIS mass loss. The FW fluxes from the five remaining AIS ocean basins all remain constant for the duration of the simulation.

In the GrIS simulation, AIS FW fluxes are held to the same constant value as in the control simulation while the GrIS FW fluxes increase (solid lines in Figure 1). Although CESM2 has the capacity to actively model the GrIS (Noël et al., 2020), we take the same approach here for overriding the default FW fluxes as in the AIS simulation to generate comparable output. For the entire 1992–2,100 simulation period, GrIS FW fluxes for each basin follow exponential curves fit to the CESM2 GrIS FW output (ensemble member 1) generated for CMIP6 (Danabasoglu et al., 2020). The GrIS‐integrated, total FW discharge increases from 1088 Gt $y^{-1}$ in 1992–2,868 Gt $y^{-1}$ in 2,100 while the total solid‐to‐liquid ratio decreases from 52%–48% to 8%–92% (Figure 1). Because the solid and liquid discharge from the GrIS follow distinct seasonal cycles, these fluxes are distributed differently in time (Figure S4 in Supporting Information S1). Subsequently, we differentiate the solid and liquid fluxes in the GrIS simulation. The drastic change in solid‐to‐liquid ratio over time accounts for the severely diminished solid ice fluxes as the GrIS ablation zone retreats further inland, reducing the ice‐ocean interface (Lenaerts et al., 2019). As mass loss is more ubiquitous throughout the ablation zone along the GrIS periphery than that of the AIS, the FW fluxes are applied around the entire GrIS coast and have disparate rates of solid and liquid discharge change across basins (Figures S2–S3, Table S2 in Supporting Information S1). As with the AIS, the FW is applied to the coastal surface grid cells and area‐weighted such that each grid cell is contributing equal amounts of total FW.

Lastly, in the AGrIS simulation, we override both ice sheets' default mass threshold, instead applying the increasing FW forcing, detailed above, for each IS simultaneously. The response of the AIS, GrIS, and AGrIS simulations, then, is directly comparable.

### 2.2 Quantifying Linear and Nonlinear Impacts From Added Freshwater

We quantify the relative strength of the linear and nonlinear response of a given scalar global climate diagnostic d to the single‐ and combined‐IS FW input using the four simulations. For our analyses, we use ${\text{OHC}}_{\text{ANTH}}$ and $C_{\text{ANTH}}$ as diagnostics: the variables we assess to diagnose changes to the climate system. $C_{\text{ANTH}}$—the anthropogenic carbon in the global ocean—is calculated as the difference between the total global DIC (exposed to increasing ${\text{CO}}_{2}$) and the global DIC from before humans started emitting ${\text{CO}}_{2}$ (preindustrial ${\text{CO}}_{2}$; forced with constant preindustrial (1850) atmospheric ${\text{CO}}_{2}$); two tracers carried by CESM2 (Long et al., 2021; Lovenduski et al., 2007). Ocean heat content is calculated as the specific heat capacity of water multiplied by the integrated product of the potential density and potential temperature depth profiles. To isolate the anthropogenic heat content, we take the anomaly relative to 1970 (the first simulation year), assuming that ${\text{OHC}}_{\text{ANTH}}$ was small before then (Cheng et al., 2021). In this paper, we discuss two forms of linearity: (a) the linear response of the diagnostics to increasing IS FW discharge with time and (b) the linear addition of output from two separate IS FW simulations compared to output from the combined‐IS simulation. The former is referred to as FW (non) linearity and the latter as combined‐IS (non)linearity hereafter. To quantify the imprint of ice sheet freshwater discharge on anthropogenic carbon and heat content, both ${\text{OHC}}_{\text{ANTH}}$ and $C_{\text{ANTH}}$ anomalies are calculated as relative to the 1970 global average.

For the three perturbation experiments X = AIS, GrIS, AGrIS, we define the anomaly diagnostic $d_{X}\left(t\right)$ as the difference between experiment X and the control at time t. Subsequently, as all terms vary in time, we drop ”(t)” for conciseness. We apply a 10‐year moving mean to $d_{X}$ to smooth over fast internal climate variability.

The total climate responses in the single‐IS forcing experiments are

(1) $$d_{\text{AIS}}={AIS}_{\text{linear}}+{AIS}_{\text{nonlinear}}$$

(2) $$d_{\text{GrIS}}={GrIS}_{\text{linear}}+{GrIS}_{\text{nonlinear}}.$$

The first step in the FW linearity decomposition is to perform linear regressions of the climate diagnostics, ${\text{OHC}}_{\text{ANTH}}$ and $C_{\text{ANTH}}$, against AIS and GrIS FW discharge from 1992 to 2100. This step produces the ${\text{AIS}}_{\text{linear}}$ and ${\text{GrIS}}_{\text{linear}}$ terms. The difference between the diagnostic response, $d_{X}$, and the temporal linear term, $X_{\text{linear}}$, yields the FW nonlinear term, $X_{\text{nonlinear}}$.

The combined‐IS forcing experiment AGrIS has an additional response, ${IS}_{\text{combined}}$ (combined‐IS nonlinearity), due to the nonlinear interaction of $FW_{\text{AIS}}$ and $FW_{\text{GrIS}}$

(3) $$d_{\text{AGrIS}}=d_{\text{AIS}}+d_{\text{GrIS}}+{IS}_{\text{combined}}.$$

We use this process to separate the FW nonlinearity that is intrinsic to the climate system from any nonlinear FW response to added ice sheet freshwater. The FW linear term, $X_{\text{linear}}$, represents the linear FW response of the climate system to added freshwater from a single ice sheet (e.g., if a certain FW discharge induces a given anomaly, doubling the FW input would induce double the anomaly) while the FW nonlinear term, $X_{\text{nonlinear}}$, measures the anomaly that evolves nonlinearly with increased discharge (following the previous example, where doubling the FW input would induce quadruple the anomaly). When considering the two ice sheets discharging FW simultaneously, there is additional, combined‐IS nonlinearity that arises from the complex interactions of anomalous changes caused by each individual ice sheet and is quantified in the ${\text{IS}}_{\text{combined}}$. We track the evolution of each term to measure the linear and nonlinear relative response to single‐IS FW fluxes (FW linearity) as well as to the nonlinear, combined‐IS FW fluxes (combined‐IS linearity).

### 2.3 Using Gaussian Process Regression to Disentangle Contributors to ${\text{OHC}}_{\text{ANTH}}$ and $C_{\text{ANTH}}$ Anomalies

Changes in ${\text{OHC}}_{\text{ANTH}}$ and $C_{\text{ANTH}}$ in response to added freshwater are caused by a complex interplay of changes in water‐mass properties, sea ice and circulation. Here, we identify the different driving factors in the FW linear and nonlinear ${\text{OHC}}_{\text{ANTH}}$ and $C_{\text{ANTH}}$ responses using a predictive, Gaussian Process Regression (GPR) model in MATLAB's Regression Learner toolbox. We supply five input variables as predictors: AMOC, SSS, sea surface temperature (SST), Arctic sea ice extent (${\text{SIE}}_{\text{NH}}$), and Antarctic sea ice extent (${\text{SIE}}_{\text{SH}}$). With each predictor impacting surface‐to‐depth transfer, water‐column stratification or air‐sea fluxes, these five predictors represent quantities critical for anthropogenic heat and carbon storage (Bronselaer & Zanna, 2020).

Leveraging these predictors as inputs, we analyze the normalized root‐mean‐square error (nRMSE; normalized such that the combined nRMSE of the five input variables sums to 1) from a Gaussian Process Regression (GPR) predictive model between its predicted output and the two CESM‐simulated diagnostics of interest, that is, annual mean ${\text{OHC}}_{\text{ANTH}}$ and $C_{\text{ANTH}}$ over 1992–2,100. RMSE values are normalized for each withheld variable to compare their influence over the global heat and carbon anomalies relative to one another as opposed to assess the actual predictive error of the GPR model. GPR models have been shown to improve predictions (Cai et al., 2020; Kupilik & Witmer, 2018), however, they do not directly yield information about the relative importance of each predictor. As such, we sequentially decompose our kernel function by withholding one predictor at a time and recording the subsequent nRMSE. In other words, to test the importance of AMOC on ocean heat content, for instance, we supply globally averaged SSS, SST, ${\text{SIE}}_{\text{NH}}$, and ${\text{SIE}}_{\text{NH}}$ time series as the GPR inputs with the globally averaged CESM2 ${\text{OHC}}_{\text{ANTH}}$ time series as the expected output and generate the nRMSE value between the expected output (from CESM2) and the predicted output (from the GPR model). The resulting nRMSE values for each withheld predictor, then, indicate their relative importance; withholding the most important predictor(s) generates the highest error between the predictive model and the CESM‐simulated ${\text{OHC}}_{\text{ANTH}}$ or $C_{\text{ANTH}}$.

## 3 Results

### 3.1 Model Validation

Historical (1970–1990), globally integrated ${\text{OHC}}_{\text{ANTH}}$ simulated by CESM2 is well aligned with observation‐based estimates, but regional biases persist particularly in the high latitude and subpolar North Atlantic, subpolar South Atlantic, and eastern tropical Pacific (cf. Figure S4 in Supporting Information S1, Figure 1 in Sabine et al. (2004); S5, cf. Figure 3a in Cheng et al. (2021)). Interpolated in situ observations from the World Ocean Database and the NOAA National Centers for Environmental Information (NCEI) estimate the global, upper 2,000 m ${\text{OHC}}_{\text{ANTH}}$ in 2020 to be 234 ZJ and 211 ZJ, respectively (Cheng et al., 2021). Compared to 1970, CESM2 produces a 255 ZJ global anomaly in 2020 in the upper 2,000 m. The slight overestimation in CESM2 could at least partially be explained by the absence of the warming hole in and around the Irminger Sea in the high‐latitude North Atlantic (Figure S1 in Supporting Information S1) and the broad warming patterns in the subpolar North and South Atlantic basins in the model (cf. Figure S5 in Supporting Information S1, Figure 3a in Cheng et al. (2021)). Similarly, CESM2 overestimates the negative ${\text{OHC}}_{\text{ANTH}}$ response in the eastern tropical Pacific. The results presented in Cheng et al. (2021) are relative to the 1958–1962 period which has a ∼10 ZJ higher global storage anomaly than 1970. Factoring in the slight cold bias of our selected reference year, CESM2 over estimates the global ocean heat storage anomaly by ∼35–55 ZJ. Acknowledging these discrepancies, CESM2 produces global ${\text{OHC}}_{\text{ANTH}}$ estimates in general good agreement with observation‐based estimates (Cheng et al., 2021).

As for ${\text{OHC}}_{\text{ANTH}}$, historical CESM2 $C_{\text{ANTH}}$ is in generally good agreement with reconstructed observations. Sabine et al. (2004) estimate 106 ± 17 Pg C for global 1994 $C_{\text{ANTH}}$, integrated over the entire water column. For the same year, CESM estimates 85 Pg C of globally integrated anthropogenic carbon storage, 4 Pg C below the bottom of the range given by Sabine et al. (2004). Long et al. (2021) find that CESM2 reproduces ∼75% of the observed $C_{\text{ANTH}}$, arguing that the discrepancy is the result of poor thermocline ventilation and the omission of pre‐1850 $C_{\text{ANTH}}$ in the model. The largest stores of historical $C_{\text{ANTH}}$ occur in the North Atlantic where both observed and CESM2‐simulated values range from ∼60–80 Pg C (compare Figure 1 to Sabine et al., 2004).

### 3.2 ${OHC}_{\text{ANTH}}$ and $C_{\text{ANTH}}$ Anomalies in Response to Ice Sheet Freshwater Discharge

**Figure 2:**
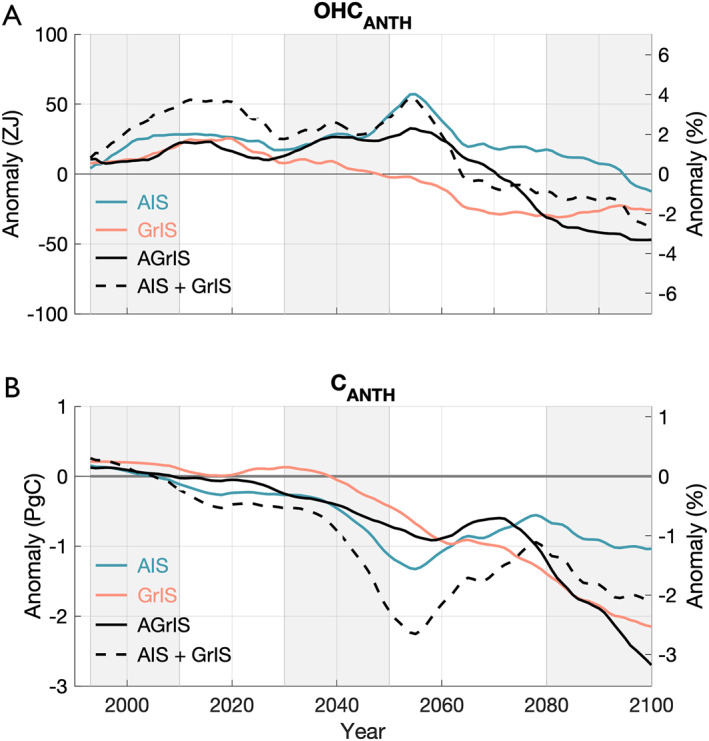
(a) Temporal evolution of global anomalous ${\text{OHC}}_{\text{ANTH}}$ (10‐year running mean) in the AIS (blue), GrIS (red), and AGrIS (black) simulations relative to the control simulation in ZJ on the left *y*‐axis and in % relative to the 1992 global control ${\text{OHC}}_{\text{ANTH}}$ value on the right *y*‐axis. Also plotted in the black dashed line is the sum of the AIS and GrIS lines. The three, gray shaded regions (1992–2010, 2030–2050, and 2080–2100) align with the three time periods examined in Table 1. (b) Same as panel (a) for $C_{\text{ANTH}}$ and in Pg (c).

By the year 2100, the global ocean stores less anthropogenic ocean heat and carbon when both ice sheets melt, but the temporal evolution of the anomalies in response to freshwater from AIS or GrIS is distinct for the two diagnostics (Figure 2, Table 1). Freshwater discharge from the Antarctic Ice Sheet anomalously increases anthropogenic ocean heat content relative to the control simulation until the year 2095 while freshwater discharge from the Greenland Ice Sheet already anomalously decreases ocean heat content after ∼2040 (compare blue and red lines in Figure 2a). In the control simulation, ${\text{OHC}}_{\text{ANTH}}$ increases from ∼1450 ZJ in 1992 to ∼4500 ZJ in 2100 (Figure S6A in Supporting Information S1). From 1992 to 2010, anomalous ${\text{OHC}}_{\text{ANTH}}$ responds similarly in the AIS and GrIS (Figure 2a). For this period, the AIS simulation stores an additional +20 ZJ of anomalous ${\text{OHC}}_{\text{ANTH}}$ compared to +12 ZJ in the GrIS simulation (Table 1). The AIS anomaly grows to +25 ZJ during the 2030–2050 period and peaks in 2052 at 72 ZJ (blue line in Figure 2a) which corresponds to +5% of the 1992 globally averaged control ${\text{OHC}}_{\text{ANTH}}$. Averaged over the same 2030–2050 period, the GrIS anomaly decreases to +5 ZJ (red line). By the end of the simulation period from 2080 to 2100, the AIS anomaly is lower than at its peak but is still anomalously higher than the control simulation (+3 ZJ). Comparatively, anomalous ${\text{OHC}}_{\text{ANTH}}$ in the GrIS simulation declines substantially through the latter half of the $21^{\text{st}}$ century, resulting in a −26 ZJ reduction of anomalous global ${\text{OHC}}_{\text{ANTH}}$ over 2080–2100 (Table 1). We note that our quantitative results are sensitive to the length of the window; for example, the global ${\text{OHC}}_{\text{ANTH}}$ anomaly in the AIS simulation is +3 ZJ from 2080 to 2100 compared to −3 ZJ from 2090 to 2100. Further, by the end of the simulation period, AIS FW induces a negligible anomaly in anthropogenic ocean heat compared to the large negative heat anomaly caused by discharge from the GrIS (Figure 2).

**Table 1:** Global ${\text{OHC}}_{\text{ANTH}}$ and $C_{\text{ANTH}}$ Anomalies Averaged Over Each Time Period From Each of the Simulations

	OHC_ANTH_ ^a^			C_ANTH_ ^b^		
	1992–2010	2030–2050	2080–2100	1992–2010	2030–2050	2080–2100
AIS	+20	+25	+3	+0.1	−0.6	−0.9
GrIS	+12	+5	−26	+0.2	−0.1	−1.9
AGrIS	+11	+24	−43	+0.1	−0.5	−2.1

^a^ ZJ.

^b^ Pg C.

In the simulation with simultaneous freshwater discharge from the Greenland and Antarctic ice sheets (AGrIS), the temporal evolution of ${\text{OHC}}_{\text{ANTH}}$ anomalies tracks that of the Antarctic Ice Sheet in the early part of the century and of the Greenland Ice Sheet in the later part of the century (black line in Figure 2a). Averaged from 2030 to 2050, the global ocean stores 24 ZJ more heat in the AGrIS simulation than in the control simulation (2 ZJ less than the AIS simulation and 19 ZJ more than the GrIS simulation). As with the AIS and GrIS simulations, anomalous ${\text{OHC}}_{\text{ANTH}}$ trends negatively after 2050 in the AGrIS simulation (Figure 2a). The negative trend in the AGrIS simulation drives a −43 ZJ loss in anomalous global ${\text{OHC}}_{\text{ANTH}}$ over the last 20 years of the simulation (46 ZJ less than the AIS simulation and 17 ZJ less than the GrIS simulation). When considering the first 78 years (1992–2070) of the simulation period, the correlation between the AIS and AGrIS simulations is 0.79 compared to 0.06 between the GrIS and AGrIS simulations (Table S4 in Supporting Information S1). The strength of the correlation between the GrIS and AGrIS increases significantly over the final 30 years (2070–2100) to −0.46. The cumulative differences between the two single‐IS simulations' and the AGrIS simulation's global ${\text{OHC}}_{\text{ANTH}}$ anomaly changes markedly over these two periods with comparable differences for the first 78 years which shift to a 7‐fold difference in the final 30 years: 710 ZJ increases to 1260 ZJ (AIS‐AGrIS) compared to −970 ZJ decreasing to 170 ZJ (GrIS‐AGrIS, Table S4 in Supporting Information S1). Over the 108‐year time series, despite similar ${\text{OHC}}_{\text{ANTH}}$ responses, the linear sum of the heat storage anomalies in the two, single‐IS simulations (Figure 2a, black dashed line) does not match that of the combined‐IS simulation.

Freshwater discharge from the Antarctic Ice Sheet rapidly reduces anthropogenic carbon storage in the global ocean until 2050, whereas freshwater discharge from the Greenland Ice Sheet produces rapid reductions in global anthropogenic carbon storage after 2040, dropping to anomalously negative values after 2070 (Figure 2b). Freshwater discharge from the Greenland Ice Sheet produces anomalously low global anthropogenic carbon storage after 2040, which continues to decline beyond the 2070s when the response to GrIS FW exceeds that of AIS FW (Table 1B). By the middle of the simulation (2030–2050), both the AIS and GrIS simulations store anomalously less $C_{\text{ANTH}}$. During this period, the AIS simulation stores less $C_{\text{ANTH}}$ than the GrIS simulation at −0.6 Pg C compared to −0.1 Pg C, respectively (Table 1). This dynamic flips by the end of the simulation as the GrIS simulation stores −1.9 Pg of anomalous global $C_{\text{ANTH}}$; 1.0 Pg C less than the −0.9 Pg C stored in the AIS simulation (Table 1). The differences between the 2030–2050 and 2080–2100 periods are largely due to the changing AIS simulation response which, after peaking in magnitude in 2053 at −1.5 Pg C (−1.8% of the 1992 globally averaged Control $C_{\text{ANTH}}$), stabilizes at around ∼‐1.0 Pg C from 2050 to 2100 (Figure 2b). Despite early and mid‐century fluctuations in both the AIS and GrIS simulations, discharge from either ice sheet results in anomalously less storage of carbon in the global ocean compared to the Control simulation by the end of the $21^{\text{st}}$ century.

While initially resembling the response from Antarctic Ice Sheet FW, $C_{\text{ANTH}}$ in the AGrIS simulation is more analogous to that of the GrIS simulation by the end of the simulation (Figure 2b). For both the 1992–2010 and 2030–2050 periods, the AGrIS simulation response mirrors that of the AIS simulation more closely, engendering +0.1 Pg and −0.5 Pg $C_{\text{ANTH}}$ anomalies, respectively (Table 1). Like the AIS simulation, the magnitude of the AGrIS $C_{\text{ANTH}}$ anomaly decreases after peaking in the 2050s, but, unlike the AIS simulation, trends negatively after 2070, resulting in a final −2.1 Pg $C_{\text{ANTH}}$ anomaly from 2080 to 2100 (Figure 2b). Ultimately, the AGrIS simulation stores 1.2 Pg less $C_{\text{ANTH}}$ than the AIS simulation and 0.2 Pg less $C_{\text{ANTH}}$ than the GrIS simulation from 2080 to 2100 (Table 1). Comparably to anomalous heat storage, global $C_{\text{ANTH}}$ storage anomalies in the AGrIS simulation are more closely correlated to those of the AIS simulation (0.97, Table S5 in Supporting Information S1) than the GrIS simulation (0.89, Table S5 in Supporting Information S1) over the first 78 years of the simulation period while producing similar (in magnitude) cumulative differences (−11 Pg C (AIS) and +11 Pg C (GrIS), Table S5 in Supporting Information S1). The AGrIS $C_{\text{ANTH}}$ response over the final 30 years shifts to being more closely correlated to the GrIS simulation (0.99 compared to 0.87 between the AIS and AGrIS simulations, Table S5 in Supporting Information S1). As with anomalous ${\text{OHC}}_{\text{ANTH}}$, the cumulative difference between the AIS and AGrIS simulations grows significantly (to 24 Pg C) while the GrIS‐AGrIS difference shrinks to 0.5 Pg C on average from 2070 to 2100 (Table S5 in Supporting Information S1). Similar to heat storage, the linearly summed carbon storage anomalies in the AIS and GrIS simulations overshoot the AGrIS carbon response in the 2030s–2060s and undershoot the AGrIS carbon response thereafter through 2100.

**Figure 3:**
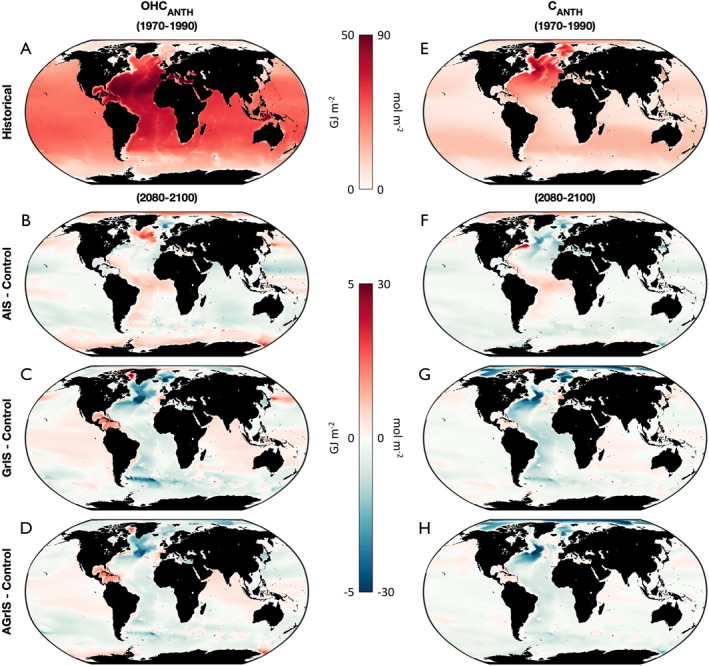
(a) Historical (1970–1990) ${\text{OHC}}_{\text{ANTH}}$ in the control simulation. (b) Anomalous ${\text{OHC}}_{\text{ANTH}}$ in the AIS simulation for the 2080–2100 period. (c) Same as panel (b) but for the GrIS simulation. (d) Same as panels (b–c) but for the AGrIS simulation. (e–h) Same as for panels (a–d) but for $C_{\text{ANTH}}$.

In addition to the distinct temporal evolution, spatial patterns differ for the uptake and storage of $C_{\text{ANTH}}$ and ${\text{OHC}}_{\text{ANTH}}$. Historically, $C_{\text{ANTH}}$ is largely stored in the North Atlantic while ${\text{OHC}}_{\text{ANTH}}$ is prevalent throughout the Atlantic Ocean in both hemispheres as detailed in Section 3.1 (1970–1990; Figures 3a and 3b). The polar oceans store little ${\text{OHC}}_{\text{ANTH}}$, historically, averaging 14 GJ $m^{-2}$ in the Arctic Ocean and 22 GJ $m^{-2}$ in the Southern Ocean south of 35°S. The strongest historical $C_{\text{ANTH}}$ signal, 87 mol C $m^{-2}$, is focused in the western North Atlantic Ocean, off the east coast of the US and Canada. The Norwegian Sea (Figure S1 in Supporting Information S1) also stores particularly high historical $C_{\text{ANTH}}$ (>70 mol C $m^{-2}$)—in good agreement with observed $C_{\text{ANTH}}$ (Section 3.1). The equatorial global ocean and high‐latitude SO south of 60°S generally lack historical $C_{\text{ANTH}}$, averaging less than 15 mol C $m^{-2}$ each. Overall, historical ${\text{OHC}}_{\text{ANTH}}$ permeates much of the global ocean while $C_{\text{ANTH}}$ storage is largely concentrated in the North Atlantic Ocean.

The responses of ${\text{OHC}}_{\text{ANTH}}$ and $C_{\text{ANTH}}$ to ice sheet melt are most disparate in the regions that are most critically important for uptake and storage (Table S3 in Supporting Information S1). By the end of the century, AIS FW engenders a positive global ${\text{OHC}}_{\text{ANTH}}$ anomaly but a negative global $C_{\text{ANTH}}$ anomaly (Figures 3b and 3f). Spatially, this difference is most prevalent in the North Atlantic around the Irminger Sea where the most extreme anomalies exceed +4 GJ $m^{-2}$ (${\text{OHC}}_{\text{ANTH}}$) and −23 Pg C $m^{-2}$ ($C_{\text{ANTH}}$). Similar positive (negative) ${\text{OHC}}_{\text{ANTH}}$ ($C_{\text{ANTH}}$) anomalies manifest in the eastern Ross Sea in the Southern Ocean (Figures 3b and 3f). Interestingly, the opposite response develops in the Gulf Stream (Figure S7 in Supporting Information S1) as a result of AIS FW wherein the region stores anomalously more $C_{\text{ANTH}}$ (+36 Pg C $m^{-2}$) but less ${\text{OHC}}_{\text{ANTH}}$ (−3 GJ $m^{-2}$) in its extremes. Overall, ${\text{OHC}}_{\text{ANTH}}$ and $C_{\text{ANTH}}$ anomalies are inversely correlated in the subpolar North Atlantic, circumpolar Southern Ocean, and Gulf Stream (Figure S8 in Supporting Information S1). Notably, the AIS FW simulation shows strong anticorrelation between heat and carbon throughout the high‐latitude Southern Ocean, especially in the AB, Ross, and Weddell Seas that is not replicated when considering GrIS FW (either alone or in tandem with AIS FW Figure S8 in Supporting Information S1). Studies have shown that different processes drive the vertical propagation of anomalous heat and carbon in the SO (Chen et al., 2019; Terhaar et al., 2021) as changes to passive transport more heavily influence carbon storage while excess heat storage patterns are dominated by a redistribution of the temperature field. Our results indicate that the addition of freshwater from ice sheets can overwhelm the temperature field redistribution signal for anthropogenic ocean heat storage and govern ${\text{OHC}}_{\text{ANTH}}$ as well. While the Antarctic FW effects on $C_{\text{ANTH}}$ storage anomalies are generally restricted to the North Atlantic, AIS‐induced ${\text{OHC}}_{\text{ANTH}}$ storage anomalies affect both North Atlantic and high‐latitude Southern Ocean heat content.

GrIS FW causes negative global anomalies in ${\text{OHC}}_{\text{ANTH}}$ and $C_{\text{ANTH}}$ which are driven in large part by the signals in the North Atlantic, high‐latitude Arctic (Figure S7 in Supporting Information S1), the Atlantic sector of the Southern Ocean, and the Norwegian Sea (Figures 3c and 3g). Unlike the AIS simulation, GrIS FW induces negative ${\text{OHC}}_{\text{ANTH}}$ responses in the subpolar North Atlantic (largest anomaly = −7 GJ $m^{-2}$), Equatorial and South Atlantic (−3 GJ $m^{-2}$, Figure S7 in Supporting Information S1), and Southern Ocean (−8 GJ $m^{-2}$) and positive responses in Baffin Bay (+5 GJ $m^{-2}$; Figure S1 in Supporting Information S1) and the Caribbean Sea (+3 GJ $m^{-2}$, Figure S7 in Supporting Information S1). The regional $C_{\text{ANTH}}$ responses to GrIS and AIS FW are more similar, particularly in the North Atlantic (−37 Pg C $m^{-2}$) and Southern Ocean (−19 Pg C $m^{-2}$). The Equatorial and South Atlantic, high‐latitude Arctic, and Gulf Stream regions stand out as developing notably incongruous $C_{\text{ANTH}}$ anomalies between the GrIS and AIS simulations. The spatial differences between AIS FW‐ and GrIS FW‐induced changes mean that globally, ${\text{OHC}}_{\text{ANTH}}$ and $C_{\text{ANTH}}$ are slightly better correlated in the GrIS simulation ($R_{\text{GrIS,global}}$ = 0.5, $R_{\text{AIS,global}}$ = 0.4), but the Irminger Sea and eastern Ross Sea still both stand out as regions where ${\text{OHC}}_{\text{ANTH}}$ and $C_{\text{ANTH}}$ anomalies are anti‐correlated (Figure S8a‐S8B in Supporting Information S1). The response of both heat and carbon to AIS discharge show inflection points at 2050, when AIS discharge begins to increase nonlinearly.

The spatial realization of ${\text{OHC}}_{\text{ANTH}}$ and $C_{\text{ANTH}}$ anomalies in the AGrIS simulation is more similar to that of the GrIS simulation than the AIS simulation. ${\text{OHC}}_{\text{ANTH}}$ and $C_{\text{ANTH}}$ anomalies from GrIS FW develop similarly throughout much of the globe in the AGrIS simulation. Notable exceptions between the anomalous ${\text{OHC}}_{\text{ANTH}}$ and $C_{\text{ANTH}}$ responses manifest in the Norwegian Sea and high‐latitude Southern Ocean where the AGrIS simulation's ${\text{OHC}}_{\text{ANTH}}$ anomaly pattern is more AIS simulation‐like. The $C_{\text{ANTH}}$ spatial anomaly pattern in the AGrIS simulation is well correlated with that of the GrIS simulation, particularly throughout the Atlantic Ocean and high‐latitude Arctic. The AIS and AGrIS simulations produce disparate anomaly patterns in the high‐latitude Arctic and eastern Equatorial Atlantic (Figure S9A in Supporting Information S1). The globally averaged correlation between AIS and AGrIS ${\text{OHC}}_{\text{ANTH}}$ is 0.5 compared to 0.6 between the GrIS and AGrIS simulations (Figure S9a‐b in Supporting Information S1). Similarly, for $C_{\text{ANTH}}$, the globally averaged AIS‐AGrIS and GrIS‐AGrIS correlations are also 0.5 and 0.6, respectively (Figure S9c‐d in Supporting Information S1). These correlations, globally averaged, indicate that the spatial realization of both heat and carbon responses in the AGrIS simulation map more closely onto the response from added Greenlandic freshwater—particularly in the North Atlantic.

### 3.3 Contributions From Linear, Nonlinear, and Combined Ice Sheet Effects

**Figure 4:**
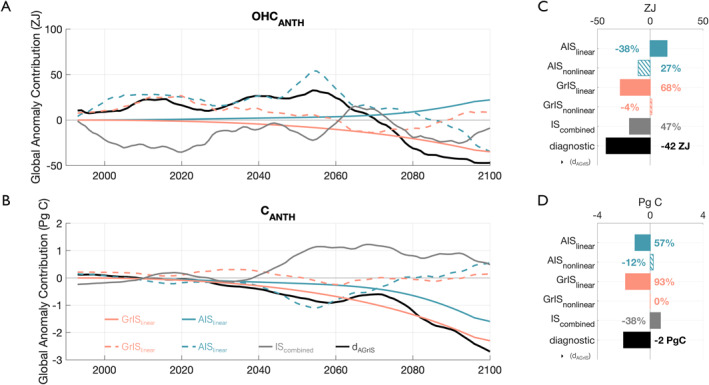
(a) Temporal evolution of each term in Equation 3 using column‐integrated ${\text{OHC}}_{\text{ANTH}}$ as the diagnostic. Solid and dashed lines show the linear and nonlinear contributions to the global ${\text{OHC}}_{\text{ANTH}}$ anomalies resulting from AIS (blue) and GrIS (red) freshwater, respectively. Gray and black lines show the combined ice sheet effects and global, column‐integrated response of ${\text{OHC}}_{\text{ANTH}}$, respectively in the AGrIS simulation. The black lines match those from Figure 2. The combined ice sheet effects are the difference between the AGrIS diagnostic response and the summed, total AIS and GrIS responses of ${\text{OHC}}_{\text{ANTH}}$; the difference between the solid and dashed black lines from Figure 2. (b) Contributions from each term in panel (a) from 2080 to 2100. Solid and hatch filled bars show the linear and nonlinear contributions from the AIS (blue) and GrIS (red). Percentages denote the relative contribution of each term to the global ${\text{OHC}}_{\text{ANTH}}$ anomaly averaged over the 2080–2100 period. The global ${\text{OHC}}_{\text{ANTH}}$ anomaly in ZJ from 2080 to 2100 is printed in black. (c–d) Same as panels (a–b) but for $C_{\text{ANTH}}$ and in Pg (c).

The temporal evolution of global ${\text{OHC}}_{\text{ANTH}}$ anomalies relative to the control simulation in response to the simultaneous freshwater discharge from the Antarctic and Greenland Ice Sheets is driven by a complex interplay of FW linear and nonlinear contributions from AIS and GrIS FW (Figure 4a). By the end of the century, the linear AIS FW contribution is the largest contributor to the global ${\text{OHC}}_{\text{ANTH}}$ (solid blue line), but is offset by the nonlinear AIS FW contribution (dashed blue line) (Figure 4a). The largest ice sheet FW contribution to anomalous ${\text{OHC}}_{\text{ANTH}}$ at any point in the simulation period is that of the nonlinear AIS FW term (dashed blue line), which reaches a maximum strength of +54 ZJ of anomalous ${\text{OHC}}_{\text{ANTH}}$ in 2055 (Figure 4a). On average from 1992 to 2055, the GrIS linear FW term contributes −3 ZJ to anomalous ${\text{OHC}}_{\text{ANTH}}$ compared to the AIS linear FW term which contributes +1 ZJ averaged over the same period. All terms but the linear AIS FW contribution decrease over the course of the simulation period, which drive the trend in the overall AGrIS simulation ${\text{OHC}}_{\text{ANTH}}$ response (solid black line; $d_{\text{AGrIS}}$ in Equation 3). These four terms—${\text{AIS}}_{\text{nonlinear}}$, ${\text{GrIS}}_{\text{linear}}$, ${\text{GrIS}}_{\text{nonlinear}}$, and ${\text{AIS}}_{\text{nonlinear}}$ (solid gray line)—overwhelm the anomalously positive ${\text{AIS}}_{\text{linear}}$ contribution in 2070 and ultimately lead to the −42 ZJ global ${\text{OHC}}_{\text{ANTH}}$ storage anomaly in the AGrIS simulation over the 2080–2100 period (Figure 4a). The leading term driving the overall negative storage anomaly is the nonlinear AIS FW term. However, considering this term together with its linear FW counterpart, the total 2080–2100 response to added AIS FW is 5 ZJ of additional heat stored in the global ocean (Figure 4b). The FW linear and nonlinear GrIS responses contribute two thirds of the overall −42 ZJ global heat storage anomaly at −28 ZJ and +2 ZJ, respectively (Figure 4c). The nonlinear AIS FW response further promotes the negative ${\text{OHC}}_{\text{ANTH}}$ storage anomaly, contributing −11 ZJ (27% of the −42 ZJ anomaly) from 2080 to 2100. Together, the linear and nonlinear GrIS FW terms as well as the nonlinear combined‐IS response contribute 89% of the total, 2080–2100 ${\text{OHC}}_{\text{ANTH}}$ as the two AIS FW terms, then, largely cancel out.

Unlike heat, global ocean anthropogenic carbon storage exhibits a limited nonlinear response to the simultaneous freshwater discharge from the Antarctic and Greenland Ice Sheets (combined‐IS nonlinearity; Figure 4b). From the beginning of the simulation, both FW linear terms (solid colored lines) contribute negatively to the storage of anomalous global $C_{\text{ANTH}}$ in the AGrIS simulation (Figure 4b). The nonlinear combined ice sheet term (solid gray line) contributes positively after ∼2040 (Figure 4b). The two FW nonlinear terms (dashed colored lines) are fairly negligible by the end of the simulation (Figure 4b). Averaged from 2080 to 2100, the global ocean in the AGrIS simulation stores 2 Pg C less than in the control simulation and is mostly driven by the linear AIS and GrIS FW effects (solid colored lines, Figure 4d). The linear GrIS FW term is the largest contributor to global $C_{\text{ANTH}}$ in the AGrIS simulation at −1.9 Pg C (93%) over the 2080–2100 period (solid red bar in Figure 4d). The linear AIS FW response enhances that of GrIS FW, contributing −1.1 Pg C (57%) to the total $C_{\text{ANTH}}$ storage anomaly (Figure 4d). While relatively important for ${\text{OHC}}_{\text{ANTH}}$ storage, the nonlinear AIS FW effects are the least important for $C_{\text{ANTH}}$, contributing +0.2 (−12%) to the global anomaly (Figure 4d). The GrIS nonlinear FW term is negligibly weak (−0.0 Pg C; 0%) compared to its linear FW counterpart by the end of the simulation (2080–2100; Figure 4d). While the global $C_{\text{ANTH}}$ anomaly in the AGrIS simulation is dominated by the two FW linear, single ice sheet terms (${\text{AIS}}_{\text{linear}}$ and ${\text{GrIS}}_{\text{linear}}$), their cumulative −3.0 Pg storage anomaly is dampened by the combined ice sheet effects (Figure 4d). The combined ice sheet FW fluxes result in a +0.8 Pg $C_{\text{ANTH}}$ storage anomaly which constitutes a −38% contribution to the global $C_{\text{ANTH}}$ response (Figure 4d).

### 3.4 Response Predictors

**Figure 5:**
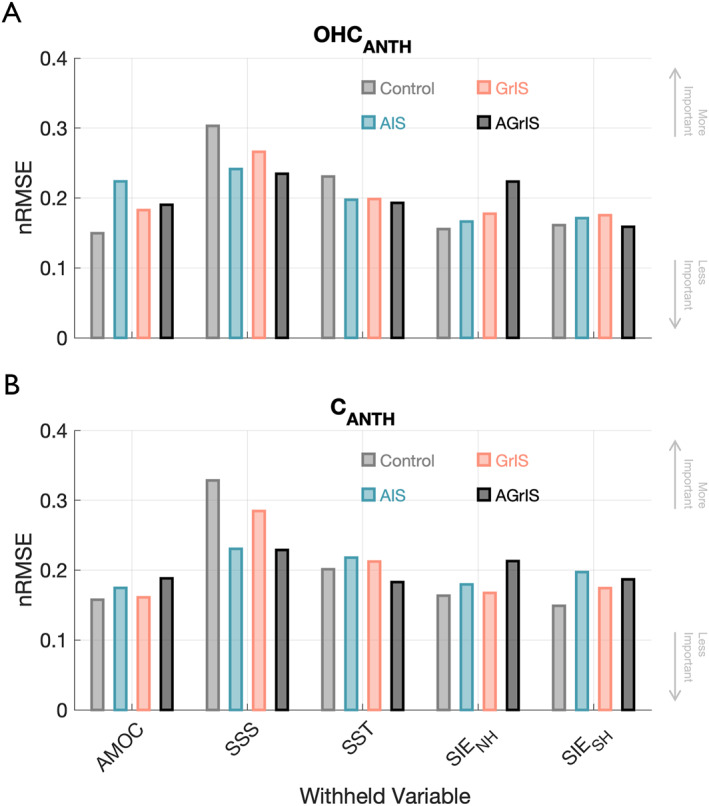
(a) Normalized Root Mean Square Error (nRMSE) values between the Gaussian Predictor Response (GPR) predicted ${\text{OHC}}_{\text{ANTH}}$ and actual ${\text{OHC}}_{\text{ANTH}}$ generated by withholding one predictor at a time for the Control (gray), AIS (blue), GrIS (pink), and AGrIS (black) simulations. (b) Same as for panel (a) but for $C_{\text{ANTH}}$.

The most important variable in predicting ice sheet‐driven changes for the 1992–2100 time period is the same for both ${\text{OHC}}_{\text{ANTH}}$ and $C_{\text{ANTH}}$: sea surface salinity (Figure 5). When systematically removing one predictor and rerunning the GPR model for each of the five predictor variables (AMOC, SSS, SST, ${\text{SIE}}_{\text{NH}}$, and ${\text{SIE}}_{\text{SH}}$), SSS produces the highest nRMSE values for both ${\text{OHC}}_{\text{ANTH}}$ and $C_{\text{ANTH}}$ for all four simulations, establishing SSS as the predominant predictor for the GPR model's predictions. For ${\text{OHC}}_{\text{ANTH}}$, normalized RMSE values when withholding SSS range from 0.025 to 0.026 across model experiments compared to 0.023 to 0.029 for $C_{\text{ANTH}}$ (Table S6 in Supporting Information S1). The next most important predictor is SST with normalized nRMSE values averaging to 0.020 across simulations for both diagnostics, global anthropogenic heat and carbon. AMOC, ${\text{SIE}}_{\text{NH}}$, and ${\text{SIE}}_{\text{SH}}$, respectively, follow in importance for ${\text{OHC}}_{\text{ANTH}}$ and are all more variable across simulations than either SSS or SST (Figure 5a, Table S6 in Supporting Information S1). For $C_{\text{ANTH}}$, ${\text{SIE}}_{\text{NH}}$ and ${\text{SIE}}_{\text{SH}}$ are the next two most important predictors with approximately equal normalized nRMSE averages followed lastly by AMOC (Figure 5b, Table S6 in Supporting Information S1).

Comparing between simulations, the nRMSE values are most varied in the Control and GrIS simulations as SSS is the most important predictor for both ${\text{OHC}}_{\text{ANTH}}$ and $C_{\text{ANTH}}$. For the AIS and AGrIS simulations, the total variation in predictor importance is within 0.01 for nRMSE values for both diagnostics. In conjunction with our earlier findings from Figure 3 and Figure S8 in Supporting Information S1, these results imply that complex processes of the SO convolute heat and carbon uptake by the ocean in the cases with elevated AIS discharge. When the GrIS discharges freshwater, SSS dominates both ${\text{OHC}}_{\text{ANTH}}$ and $C_{\text{ANTH}}$ signals indicating a pervasive modulation of North Atlantic uptake by the GrIS.

Sea surface salinity as the most influential predictor in anomalous ocean carbon storage aligns well with results from Chen et al. (2019) and Terhaar et al. (2021) wherein the propagation of surface $C_{\text{ANTH}}$ to depth is slowed due to the freshened surface and strengthened density gradient (Bourgeois et al., 2022; Moorman et al., 2020; Sadai et al., 2020; Swingedouw et al., 2015). Interpreting the heightened influence of AMOC over ${\text{OHC}}_{\text{ANTH}}$ is more complex. We posit that AMOC's influence over ${\text{OHC}}_{\text{ANTH}}$ could be a result of the weakening of AMOC which analysis of the CESM Large Ensemble suggests has been deepening a warming hole in the North Atlantic (Huguenin et al., 2022; Yeager et al., 2016)—one of the most critical regions for heat uptake (Kostov et al., 2014). This answer is convoluted, though, as SSS affects the strength of AMOC (as does SST) and so extricating the individual components' impact on ocean heat storage (and ocean carbon storage) could be refined by additional sensitivity experiments.

### 3.5 Timing and Depth‐Distribution of Combined Ice Sheet Effects

**Figure 6:**
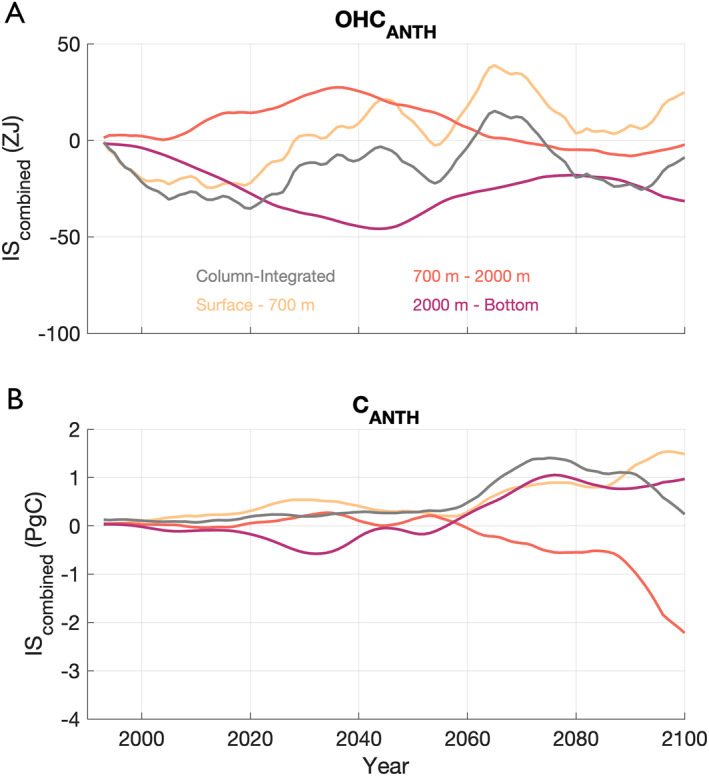
(a) Temporal evolution of the ${IS}_{\text{combined}}$ term (see Equation 3) for ${\text{OHC}}_{\text{ANTH}}$ for the surface ‐ 700 m depth (yellow), 700–2,000 m depth (orange), 2,000 m ‐ bottom (pink), and integrated over the whole water column (gray). The gray lines match those from Figure 4. (b) Same as panel (a) but for $C_{\text{ANTH}}$.

Changes in global ocean heat content driven by simultaneous ice sheet melt propagate more quickly from surface to depth than ice sheet‐driven changes in anthropogenic carbon (Figure 6). Surface changes in the combined ice sheet effects on ${\text{OHC}}_{\text{ANTH}}$ first become apparent at depth within the first 30 years of simulation time (Figure 6a). Considering ${\text{OHC}}_{\text{ANTH}}$ in the upper ocean (surface ‐ 700 m), the strength of the combined ice sheet term peaks in 2065 at 39 ZJ. The magnitude of the combined ice sheet effects on middle (700–2,000 m) and lower (2,000 m ‐ bottom) ocean layers also reach maxima in the middle decades of the $21^{\text{st}}$ century at +27 ZJ in 2036 (middle) and −46 ZJ in 2044 (lower; Figure 6a). In contrast, the combined ice sheet impacts are not realized until after 2070 for $C_{\text{ANTH}}$ for all depth levels (Figure 6b). The magnitude of combined ice sheet effects on $C_{\text{ANTH}}$ in the upper, middle, and lower ocean layers peak in 2097, 2100, and 2076 at 1.5 Pg C, −2.2 Pg C, and 1.0 Pg C, respectively. From 2080 to 2100, the largest ${\text{OHC}}_{\text{ANTH}}$ anomalies from combined ice sheet effects manifest in the bottom ocean layer (−24 ZJ) while largest $C_{\text{ANTH}}$ anomalies manifest in the middle ocean layer (−1.1 Pg C). Furthermore, the lower ocean layer stores anomalously less ${\text{OHC}}_{\text{ANTH}}$ (−24 ZJ) but more $C_{\text{ANTH}}$ (+0.8 Pg C) than the control over the same 2080–2100 period. In both, however, the net impacts from integrating over the column are such that the combined ice sheet contributions are positive by 2100 (Figure 6).

## 4 Discussion and Conclusions

We leverage a state‐of‐the‐art GCM experiment with comparably applied AIS and GrIS FW forcings to directly contrast the ${\text{OHC}}_{\text{ANTH}}$ and $C_{\text{ANTH}}$ impacts generated by each ice sheet's projected mass changes. We offer a new perspective on quantifying the temporal linear and nonlinear contributions to ${\text{OHC}}_{\text{ANTH}}$ and $C_{\text{ANTH}}$ due to FW from each ice sheet separately as well as the nonlinearity that arises from both ice sheets' combined discharge. By the end of the $21^{\text{st}}$ century, the combined effect of both ice sheets is an anomalous reduction in both ${\text{OHC}}_{\text{ANTH}}$ and $C_{\text{ANTH}}$ storage in the global ocean. The global ${\text{OHC}}_{\text{ANTH}}$ anomaly in the combined ice sheet scenario generally follows the positive trend of the AIS simulation through the first half of the $21^{\text{st}}$ century and the negative trend of the GrIS simulation through the second half. Freshwater discharge from each individual ice sheet induces a negative $C_{\text{ANTH}}$ trend, and, as with ${\text{OHC}}_{\text{ANTH}}$, the combined ice sheet response follows the positive trend response realized in the AIS simulation more closely through roughly 2050 and the GrIS simulation thereafter. The high‐latitude North Atlantic and Southern Ocean—important regions for historical anthropogenic heat and carbon storage and fluxes (Bronselaer & Zanna, 2020; Gruber et al., 2002, 2019; Huguenin et al., 2022)—develop disparate anomalous ${\text{OHC}}_{\text{ANTH}}$ and $C_{\text{ANTH}}$ responses to ice sheet FW. Diagnosing the disparate responses of ${\text{OHC}}_{\text{ANTH}}$ and $C_{\text{ANTH}}$ across the two hemispheres requires additional analysis to explore the role of discharge on changes in, for example, AMOC, sea ice extent, atmospheric teleconnections, and the Antarctic Circumpolar Current. Anomalies in both global ${\text{OHC}}_{\text{ANTH}}$ and $C_{\text{ANTH}}$ respond nonlinearly both to individual ice sheet freshwater discharge (FW nonlinearity) as well as simultaneous ice sheet freshwater discharge (combined‐IS nonlinearity). The linear response to GrIS FW dominates both anthropogenic ocean heat and carbon signals. Despite distinct realizations, SSS is the preeminent driver of global, ice sheet‐induced changes to both ${\text{OHC}}_{\text{ANTH}}$ and $C_{\text{ANTH}}$. Manifesting disparately in both depth and time, global ${\text{OHC}}_{\text{ANTH}}$ anomalies develop more quickly than global $C_{\text{ANTH}}$ anomalies. As SSS is an important factor for controlling stratification and, hence, downward transfer of anthropogenic heat and carbon, it seems probable that salinity‐driven changes in stratification are affecting the propagation of heat and carbon and, thus, further uptake (Bourgeois et al., 2022; Terhaar et al., 2020).

Stemming from distinct historical storage, disparate ${\text{OHC}}_{\text{ANTH}}$ and $C_{\text{ANTH}}$ changes are further accentuated by their divergent responses to ice sheet FW. As a region of markedly high uptake for both ${\text{OHC}}_{\text{ANTH}}$ and $C_{\text{ANTH}}$, contrasting responses in the high‐latitude North Atlantic demonstrate that changes to one diagnostic do not necessarily directly correspond to changes in the other. Bronselaer and Zanna (2020) also explore the future relationship of ${\text{OHC}}_{\text{ANTH}}$ and $C_{\text{ANTH}}$ resulting from changing atmospheric conditions. Instead of directly investigating changing FW fluxes from ice sheets, they analyze output from two comparable simulations: one that regulates ocean currents to that of the preindustrial state and another that is allowed to evolve freely under transient, 1% $y^{-1}$ increase in anthropogenic carbon (Bronselaer & Zanna, 2020). In their study, they find a linear relationship between the global ocean ${\text{OHC}}_{\text{ANTH}}$ and $C_{\text{ANTH}}$ uptake due to anthropogenic changes (Bronselaer & Zanna, 2020). In contrast, our results explore the anomalous changes to these global inventories owing solely to differences stemming from increasing freshwater fluxes from the Antarctic and Greenland Ice Sheets.

The reduced $C_{\text{ANTH}}$ signal in the AGrIS simulation—driven by GrIS FW—indicates that the high‐latitude North Atlantic, and, thus, the global ocean, will do less to mitigate rising atmospheric carbon levels over the coming century. The global $C_{\text{ANTH}}$ anomaly in the AGrIS simulation (−2.1 Pg C) represents a 2.5% change to the global $C_{\text{ANTH}}$ inventory in the control simulation in 1992 (∼0.5% of the total inventory in 2100). Weakening the ocean's ability to store additional $C_{\text{ANTH}}$ will lead to elevated atmospheric ${\text{CO}}_{2}$ concentrations by the end of the $21^{\text{st}}$ century. The global ocean buffering capacity for increasing atmospheric temperatures is also diminished (−3%) as a result of combined ice sheet FW fluxes—particularly in the North Atlantic, a region that helps govern global ${\text{OHC}}_{\text{ANTH}}$ trends (Gruber et al., 2002; Huguenin et al., 2022). In constellation, positive and negative global storage anomalies in the upper ocean (0–700 m) and middle ocean (700–2,000 m), respectively, indicate that less ${\text{OHC}}_{\text{ANTH}}$ and $C_{\text{ANTH}}$ are being transported to depth; instead accumulating in the surface layer. The relatively small difference in global $C_{\text{ANTH}}$ storage indicates that the strength of the warming scenario is a significantly stronger driver of anthropogenic carbon in the ocean than ice sheet runoff. As such, it is plausible that these results would be exacerbated under stronger warming conditions and/or over a longer investigation period. Thus, as the surface ocean stores more ${\text{OHC}}_{\text{ANTH}}$ and $C_{\text{ANTH}}$ under this strong atmospheric warming, it is less capable of taking up more (Gruber et al., 2023; Maier‐Reimer & Hasselmann, 1987), indicating a further reduction in uptake efficacy for both parameters beyond 2100.

The effects of singular ice sheet freshwater discharge on anthropogenic ocean heat and carbon storage do not linearly combine to produce the effects of simultaneous ice sheet freshwater discharge. To project realistic FW‐induced changes, simulating both ice sheets simultaneously is thus imperative. Summing the FW linear components of the globally averaged anomalies from the AIS and GrIS simulations leads to a 46 ZJ mismatch of anomalous ${\text{OHC}}_{\text{ANTH}}$ storage—5 ZJ added heat anomaly compared to the −42 ZJ heat anomaly of the AGrIS simulation. For $C_{\text{ANTH}}$ storage, the sum of the linear single‐IS FW terms results in a significant overestimation of $C_{\text{ANTH}}$ storage (1.1 Pg C) when compared to the AGrIS simulation. As modeling centers move toward incorporating active ice sheet components into their GCMs, first focusing on representing increasing GrIS FW fluxes is critical for estimating the projected the global and regional FW‐induced changes to both ${\text{OHC}}_{\text{ANTH}}$ and $C_{\text{ANTH}}$.

However, AIS FW impacts are still robust enough to affect the global ${\text{OHC}}_{\text{ANTH}}$ and $C_{\text{ANTH}}$ inventories, the eventual inclusion of an active AIS is also imperative for getting an accurate assessment of these changes. Because these AIS FW impacts manifest later than those from GrIS FW, shorter simulations will indicate higher GrIS dependence in the evolution of global ${\text{OHC}}_{\text{ANTH}}$ and $C_{\text{ANTH}}$. Li, Marshall, et al. (2023), who use linear convolution theory to disentangle linear and nonlinear combined‐IS responses, find the AIS FW causes a stronger response in air temperature, sea ice extent and deep‐water formation and that these responses only become nonlinear after exceeding a 5000 Gt $y^{-1}$ melt rate threshold. Instead of a slow ramp up of FW discharge, Li, Marshall, et al. (2023) apply a step‐wise increase of FW from 0 Gt $y^{-1}$ to 500 Gt $y^{-1}$, 2000 Gt $y^{-1}$, and 5000 Gt $y^{-1}$ for each individual ice sheet as well as their combined ice sheet simulation. Unlike their experiment, we gradually increase spatially heterogeneous ice sheet FW fluxes and find that the GrIS rather than the AIS dominates anomalous changes through the $21^{\text{st}}$ century. Moreover, we find that the combined ice sheets' nonlinear impacts on ${\text{OHC}}_{\text{ANTH}}$ and $C_{\text{ANTH}}$ anomalies begin to manifest in the 2050s, well before FW fluxes from either ice sheet exceed 5000 Gt $y^{-1}$. That said, due to the longer time‐scale of the realization of AIS FW impacts, longer simulations will be necessary to fully quantify the cumulative AIS FW impacts as the AIS becomes an active component in GCMs. Future investigations can further clarify the mechanisms driving the anomalous heat and carbon signals to freshwater input, for example, akin to Chen et al. (2019) and Bronselaer and Zanna (2020).

The major caveats of this work include the use of a single ensemble member and the application of the added FW fluxes to the ice sheet‐adjacent ocean grid cells. Additionally, our approach to projecting future FW fluxes assumes that past mass loss spatial patterns will map onto future mass loss spatial patterns. Our AIS FW forcings assume that past spatial patterns of mass loss will continue into the future. Our observational record of mass change for both ice sheets extends back only ∼20 years, restricting our ability to assess both ice sheet‐integrated and spatially resolved decadal and interdecadal trends. Supplementing these data with information from ice sheet models indicates that past WAIS mass loss is projected to not only continue, but intensify in the future (Rignot et al., 2019; van den Akker et al., 2024). Our results may be affected by the spatial distribution of the added AIS discharge but to fully assess the influence of the FW placement will require additional analysis. The future AIS‐integrated FW forcing lacks important climate feedback as it subsumes output generated by a model without an active AIS component. Future GrIS FW fluxes are based on active Greenland CESM2 model output simulated under SSP5‐8.5 atmospheric forcing. The AIS FW flux values are predicated upon the assumption of ice shelf mass balance which we made for two reasons: (a) the GRACE satellites do not measure mass changes of the ice shelves and (b) CESM2 currently lacks the ability to model floating ice shelves. The former reason means that we have little information to guide any ice shelf mass imbalances and the latter reason means that, even if we did have ice shelf mass imbalance estimates, we are not yet able to simulate them in CESM2. As a result, we assume the ice shelves are in mass balance and that mass changes to the grounded AIS are realized immediately as FW fluxes into the surrounding ocean. Finally, as these FW fluxes (modeled as salinity fluxes with no information on temperature or momentum) are distributed into the surrounding ocean, we apply them directly to the surface coastal grid cells. Realistically, calved ice distributes FW solely to the surface ocean but is spread further offshore as it is carried via ocean currents while basal melt is distributed horizontally across the underside of the ice shelves, at depths exceeding 1 km (Dinniman et al., 2016). Another important limitation of this work is the examination of a single ensemble member for each of the four discharge regimes (Control, AIS, GrIS, and AGrIS). The use of a single ensemble member restricts our ability to quantify the influence of internal variability on our results and we acknowledge that this variability could impact our assessment of non‐linearity in the response of ocean heat and anthropogenic carbon to freshwater forcing. Future modeling experiments will help contextualize our results by examining how much influence internal variability has on the sign and magnitude of anomalous ocean heat and carbon content changes caused by ice sheet discharge.

Despite these limitations, our findings underscore the need for further exploration of ice sheet FW impacts on global anthropogenic heat and carbon storage. The AIS and GrIS engender distinct responses for global ${\text{OHC}}_{\text{ANTH}}$ and global $C_{\text{ANTH}}$ anomalies and their impacts cannot simply be linearly added to capture their combined effects. As the polar regions are uniquely important for heat and carbon uptake, actively modeling the ice sheets and incorporating feedbacks induced by their melt will impact projections of the ocean's capacity to mitigate rising atmospheric carbon and heat.

## Supporting information

Supporting Information S1

## Data Availability

Data from the CONTROL simulation presented in this paper are publicly available at Gorte et al. (2024e) (historical) and Gorte et al. (2024f) (2015–2100). Data from the AIS simulation presented in this paper are publicly available at Gorte et al. (2024c) (historical) and Gorte et al. (2024d) (2015–2100). Data from the GrIS simulation presented in this paper are publicly available at Gorte et al. (2024g) (historical) and Gorte et al. (2024h) (2015–2100). Data from the AGrIS simulation presented in this paper are publicly available at Gorte et al. (2024a) (historical) and Gorte et al. (2024b) (2015–2100).
