# Supplementary material for: The Nonlinear and Distinct Responses of Ocean Heat Content and Anthropogenic Carbon to Ice Sheet Freshwater Discharge in a Warming Climate
Source: Earths Future. 2024 Nov 24;12(11):e2024EF004475. doi: 10.1029/2024EF004475 (PMC11586472; doi:10.1029/2024EF004475)
Supplement: Supplementary file 1 — Supporting Information S1 [file EFT2-12-0-s001.pdf]

Supporting Information for The nonlinear and distinct responses of ocean heat content and anthropogenic carbon to ice sheet freshwater discharge in a warming climate

Tessa Gorte[1,2], Nicole S. Lovenduski[1,2], Cara Nisssen [1,2], Jan T. M. Lenaerts[1], Jeffrey B. Weiss[1]

[1]Department of Atmospheric and Oceanic Sciences, University of Colorado Boulder, Boulder CO, USA

[2]Institute of Arctic and Alpine Research, University of Colorado Boulder, Boulder CO, USA

Contents of this file

1. Figures S1 to S10
2. Tables S1 to S6

Introduction

References

Cheng, L., Abraham, J., Trenberth, K. E., Fasullo, J., Boyer, T., Locarnini, R., ... Zhu, J. (2021). Upper ocean temperatures hit record high in 2020. *Advances in Atmospheric Sciences*, *38*, 523-530. doi: 10.1007/s00376-021-0447-x

Sabine, C. L., Feely, R. A., Gruber, N., Key, R. M., Lee, K., Bullister, J. L., ... Rios, A. F. (2004). The Oceanic Sink for Anthropogenic CO_2. *Science*, *305*, 367-371. (doi: 10.1126/science.1097403) doi: 10.1126/science.1097403

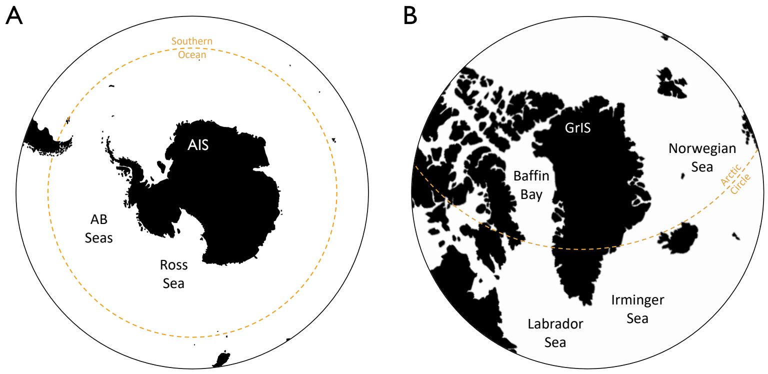

Figure S1. The Antarctic Ice Sheet (AIS, A) the surrounding Southern Ocean – containing the Amundsen and Bellingshausen Seas (AB Seas) and Ross Sea – as well as the Greenland Ice Sheet (GrIS, B) with the Norwegian Sea and Baffin Bay in the Arctic Ocean and Labrador and Irminger Seas in the North Atlantic.

Table S1. Antarctic Ice Sheet (AIS) basin-integrated total freshwater (FW) flux values for 1992 and (2100) for each simulation. All fluxes are given in units of Gt y^{-1} (1 Gt = 1 Gigaton = 10^{12} kg).

	$IO^{1992}_{(2100)}$	$PO^{1992}_{(2100)}$	$RS^{1992}_{(2100)}$	$AB^{1992}_{(2100)}$	$AP^{1992}_{(2100)}$	$WS^{1992}_{(2100)}$	$Tot.^{1992}_{(2100)}$
Control	383	606	246	716	322	502	2775
	(383)	(606)	(246)	(716)	(322)	(502)	(2775)
AIS	383	606	246	716	322	502	2775
	(383)	(606)	(246)	(7039)	(322)	(502)	(9098)
GrIS	383	606	246	716	322	502	2775
	(383)	(606)	(246)	(716)	(322)	(502)	(2775)
AGrIS	383	606	246	716	322	502	2775
	(383)	(606)	(246)	(7039)	(322)	(502)	(9098)

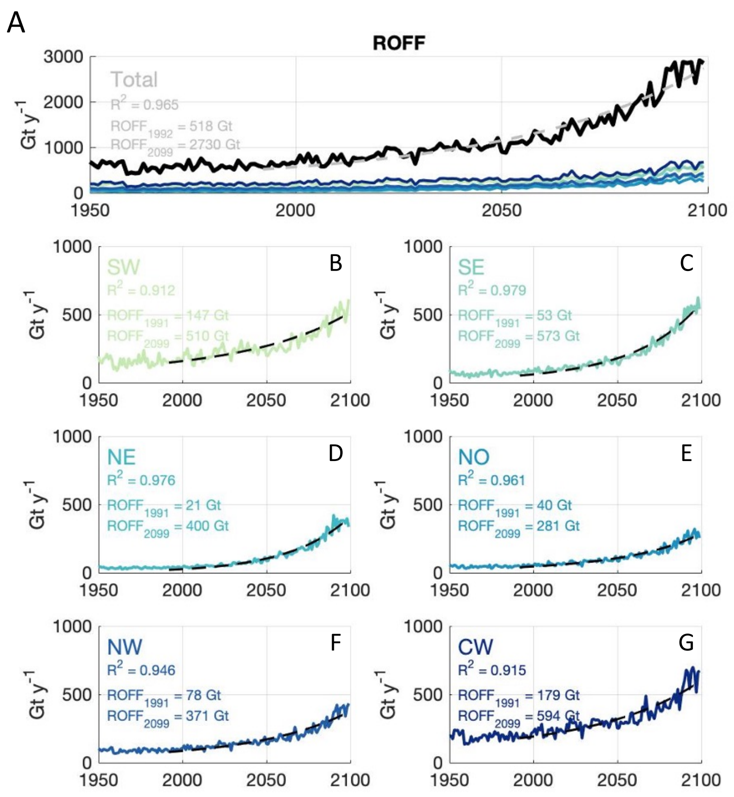

Figure S2. Liquid runoff (ROFF) CESM2 CMIP6 output time series integrated across the entire entire GrIS (A, black line), and each of its six drainage basins (colored solid lines). The gray dashed line denotes the basin-integrated freshwater (FW) forcing values applied in the GrIS and AGrIS simulations. Time series of the basin-integrated ROFF values generated by the CESM2 output (solid colored lines) and the FW forcing (black dashed lines, B-G). Also shown are the basin-integrated 1992 and 2100 values and the R^2 between the CESM2 output data and exponential fit used to guide the GrIS freshwater forcing in the corresponding colors.

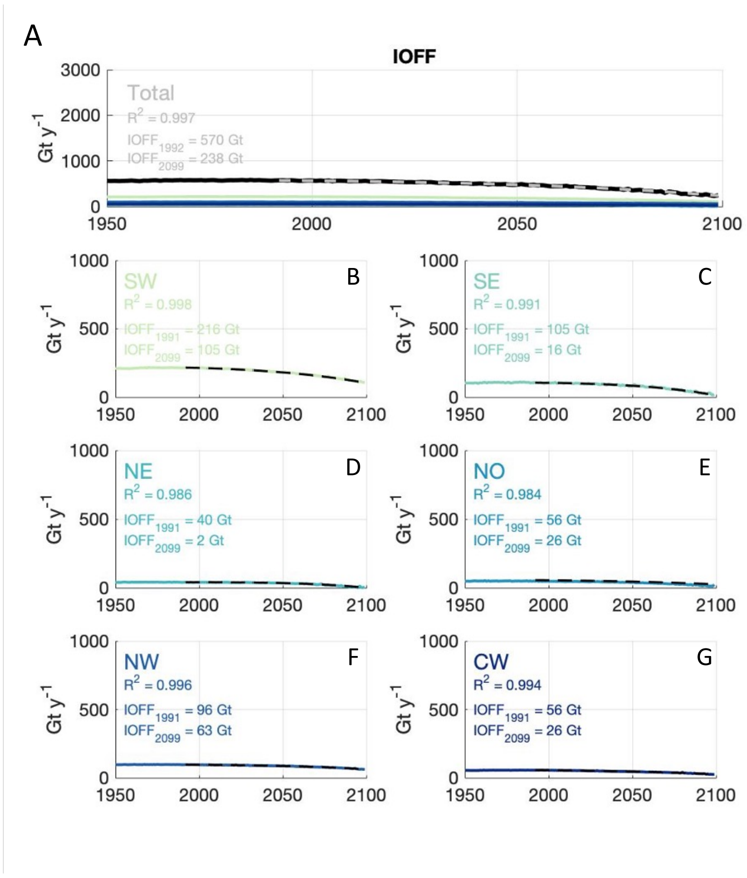

Figure S3. Same as Figure S2 for solid runoff (IOFF).

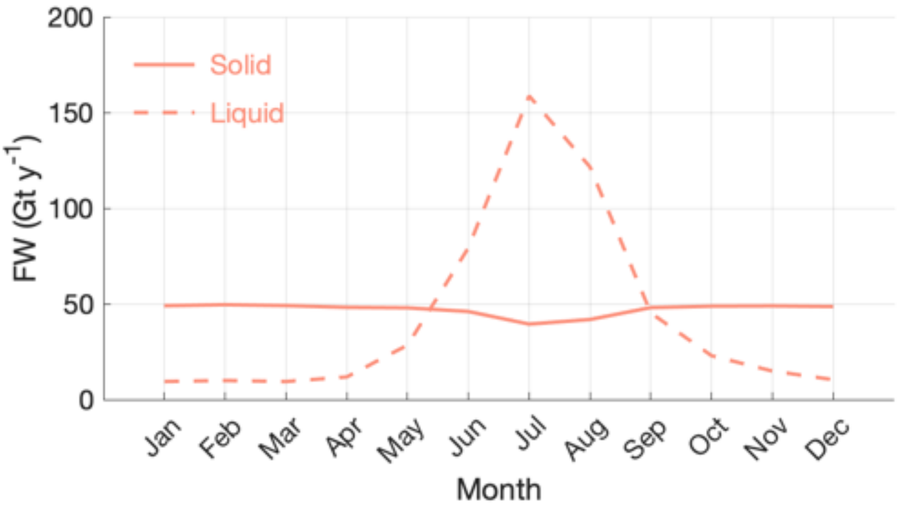

Figure S4. Solid (solid pink line) and liquid (dashed pink line) GrIS-integrated discharge annual time series for 1992.

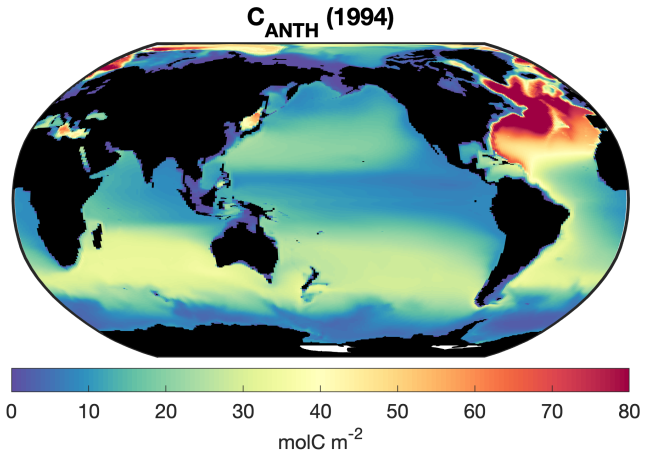

Figure S5. Control simulation column inventory of anthropogenic carbon (C_{ANTH}) in molC m^{-2} in 1994 (cf. Figure S4, Figure 1 in Sabine et al. (2004)).

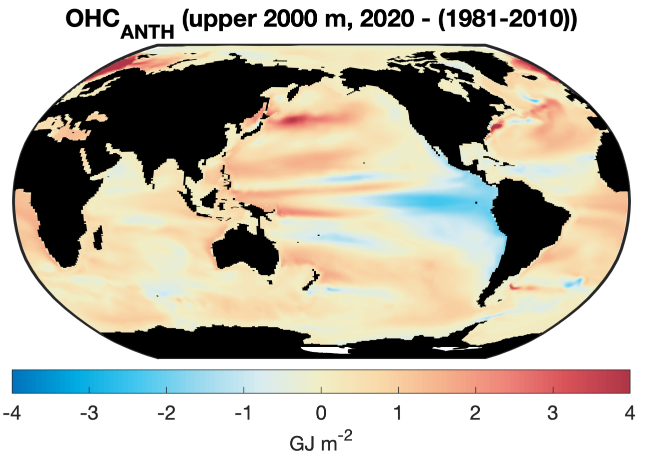

Figure S6. Control simulation ocean heat content anomaly (OHC_{ANTH}) in GJ m^{-2} in the upper 2000 m of the ocean in 2020 relative to the 1981-2010 average (cf. Figure 3(a) in Cheng et al. (2021))

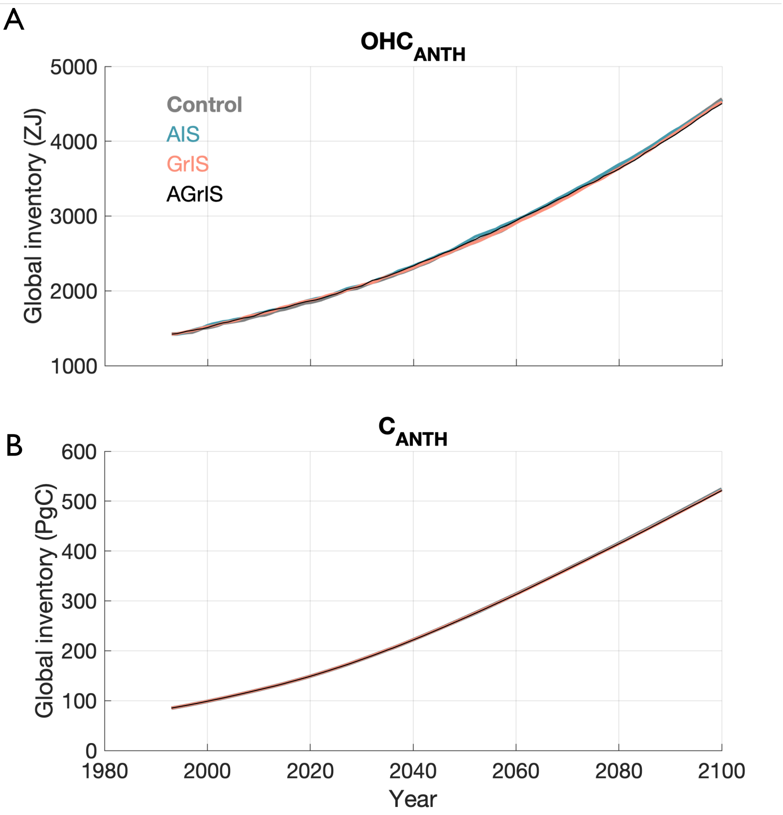

Figure S7. Column-integrated, global anthropogenic ocean heat content (OHC_{ANTH}, A) in ZJ and carbon (C_{ANTH}, B) in PgC in the Control simulation (thick gray line), AIS simulation (blue line), GrIS simulation (red line), and AGrIS simulation (black line).

Table S2. Greenland Ice Sheet (GrIS) basin-integrated total freshwater (FW) flux values for 1992 and (2100) for each simulation. All fluxes are given in units of Gt y^{-1} (1 Gt = 1 Gigaton = 10^{12} kg).

	$SW^{1992}_{(2100)}$	$SE^{1992}_{(2100)}$	$NE^{1992}_{(2100)}$	$NO^{1992}_{(2100)}$	$NW^{1992}_{(2100)}$	$CW^{1992}_{(2100)}$	$Tot.^{1992}_{(2100)}$
Control	363	158	61	196	174	235	1187
	(363)	(158)	(61)	(196)	(174)	(235)	(1187)
AIS	363	158	61	196	174	235	1187
	(363)	(158)	(61)	(196)	(174)	(235)	(1187)
GrIS	363	158	61	196	174	235	1187
	(615)	(589)	(402)	(307)	(434)	(620)	(2967)
AGrIS	363	158	61	196	174	235	1187
	(615)	(589)	(402)	(307)	(434)	(620)	(2967)

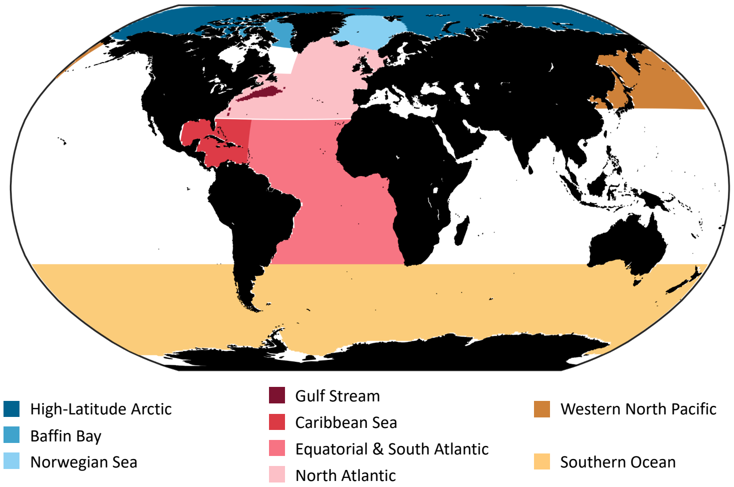

Figure S8. Important regions of global anthropogenic ocean heat content and carbon storage and change: the high latitude Arctic (dark blue), Baffin Bay (medium blue), the Norwegian Sea (light blue) in the Arctic Ocean, the Gulf Stream (dark red), Caribbean Sea (medium red), Equatorial and South Atlantic (light red), and North Atlantic (light pink) in the Atlantic Ocean, as well as the western North Pacific (brown), and Southern Ocean (yellow).

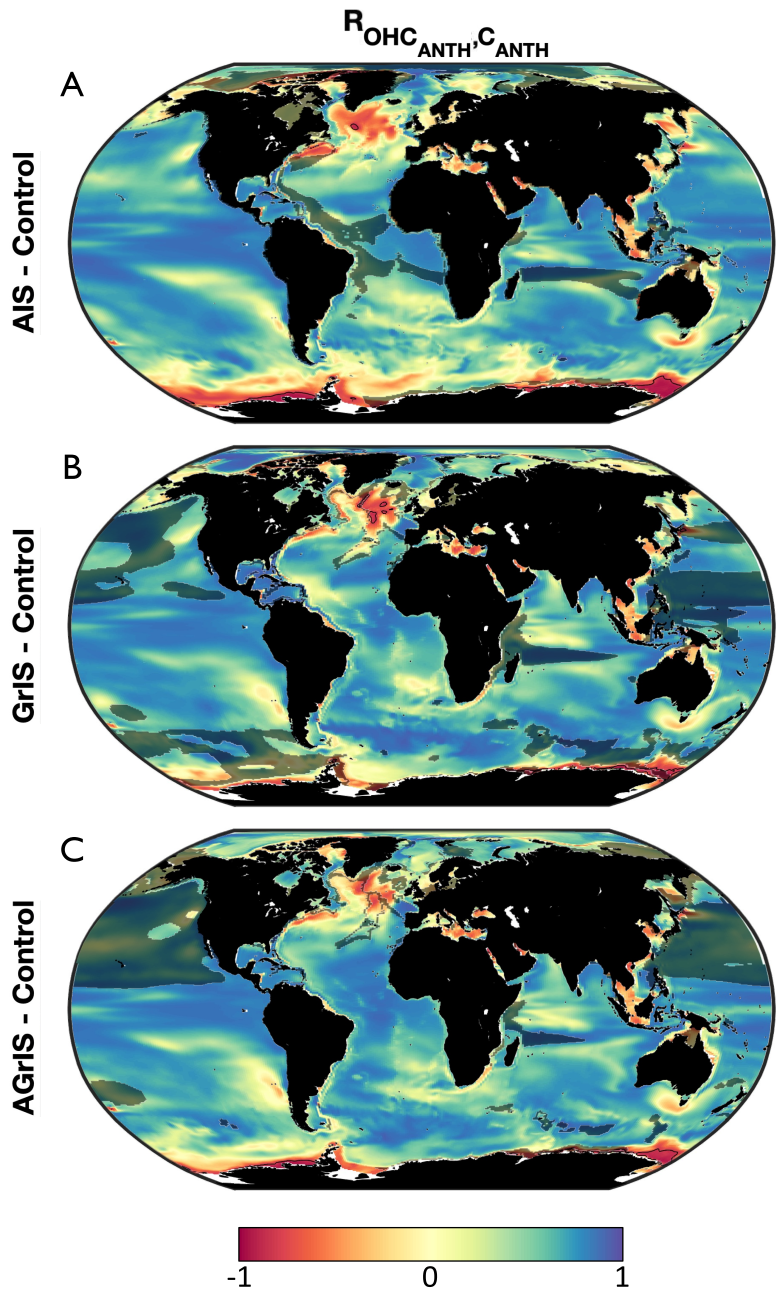

Figure S9. Linear correlation between (unsmoothed, 108-year) anomalous anthropogenic ocean heat content (OHC_{ANTH}) and carbon (C_{ANTH}) at each ocean grid cell in the AIS simulation (A), GrIS simulation (B), and AGrIS simulation (C). Dark shading indicates regions that are not statistically significant at the 95% confidence interval.

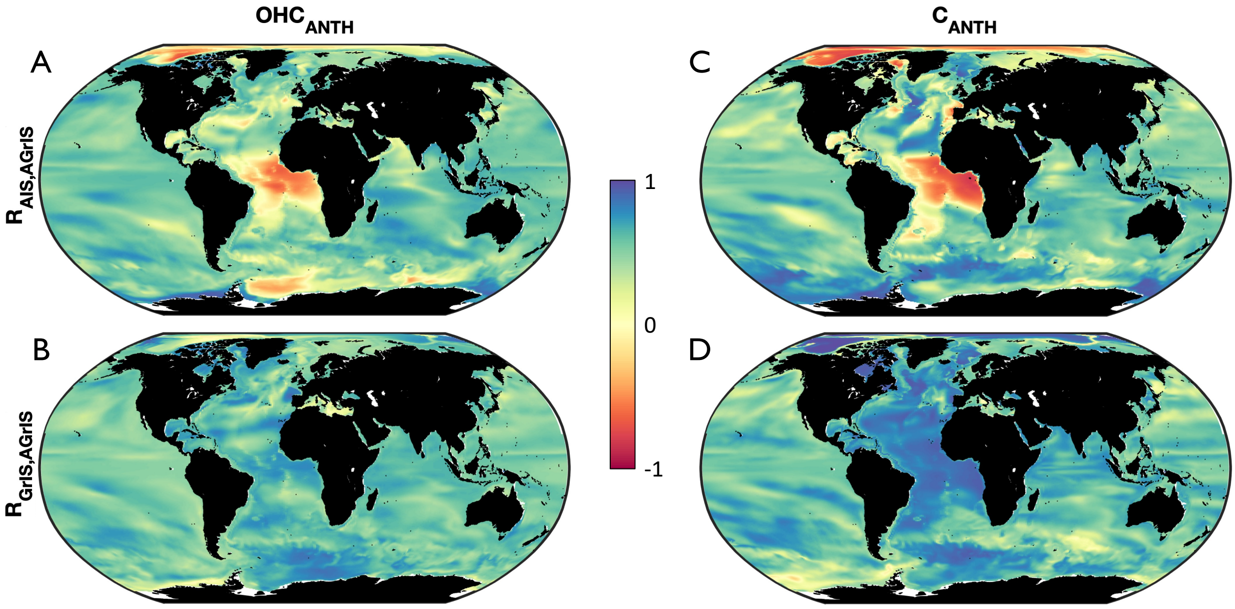

Figure S10. Linear correlation in anomalous anthropogenic ocean heat content (OHC_{ANTH}) response to ice sheet freshwater (FW) in the AIS and AGrIS simulations (A) and GrIS and AGrIS simulations (B). Same for anomalous anthropogenic carbon (C_{ANTH}, C-D).

Table S3. Global OHC_{ANTH} and C_{ANTH} maximum, mean, and minimum anomalies averaged over each time period from each of the simulations. The regions denoted in this table are outlined in Figure S7.

		$OHC^{\dagger}_{ANTH}$			$C^{\ddagger}_{ANTH}$		
		AIS	GrIS	AGrIS	AIS	GrIS	AGrIS
High-latitude Arctic	max	+2.5	+1.6	+0.6	+10.4	+14.7	+3.5
	mean	+0.4	–0.1	–0.5	+1.9	–4.2	–5.8
	min	–0.3	–2.2	–2.9	–3.3	–34.2	–35.2
Baffin Bay	max	+1.3	+4.9	+2.7	+9.3	+1.0	+0.8
	mean	–0.4	+0.7	+0.4	+0.9	–0.9	–2.1
	min	–1.6	–2.3	–2.9	–2.4	–2.4	–9.2
Norwegian Sea	max	+1.7	+1.3	+0.4	+7.8	+10.7	+4.6
	mean	–0.7	–1.4	–0.9	–4.3	–9.3	–5.7
	min	–4.1	–6.5	–2.7	–23.8	–40.9	–24.8
North Atlantic	max	+4.1	+2.6	+3.3	+10.0	+11.8	+11.0
	mean	+0.3	–1.4	–1.1	–4.6	–4.4	–4.9
	min	–3.8	–6.5	–5.5	–22.7	–36.9	–38.8
W. North Pacific	max	+2.9	+3.5	+0.3	+2.7	+7.0	+2.8
	mean	+0.3	+0.2	–0.3	–0.9	+0.3	–0.5
	min	+1.3	–2.0	–2.1	–20.6	–5.9	–9.4
Gulf Stream	max	+2.5	+2.1	+3.7	+36.0	+6.1	+8.3
	mean	+0.0	–0.8	+0.0	+15.1	–13.0	–11.2
	min	–3.1	–3.1	–2.5	+10.0	–34.7	–30.6
Caribbean Sea	max	+1.7	+3.4	+2.9	+10.9	+9.4	+8.4
	mean	–0.4	+1.1	+1.1	–0.7	–0.1	+0.6
	min	–1.1	–1.3	–2.5	–3.7	–13.7	–9.2
Eq. & S. Atlantic	max	+1.7	+3.4	+2.9	+9.1	+5.4	+4.9
	mean	+0.2	–0.4	–0.4	+1.2	–5.5	–3.9
	min	–1.7	–2.5	–2.0	–8.4	–13.1	–9.3
Southern Ocean	max	+3.8	+1.1	+3.5	+7.0	+5.5	+4.9
	mean	+0.1	–0.4	–0.2	–2.2	–1.1	–2.1
	min	–1.6	–7.8	–4.1	–20.1	–18.7	–19.9

† GJ m^{-2}
‡ molC m^{-2}

Table S4. Correlations and cumulative differences between the globally averaged OHC_{ANTH} time series between the combined ice sheet freshwater (AGrIS) and the Antarctic freshwater (AIS) simulations as well as the AGrIS and Greenlandic freshwater (GrIS) simulations calculated for the 1992-2070 and 2070-2100 periods.

	Correlation	Cumulative Difference (ZJ)	Correlation	Cumulative Difference (ZJ)
	(1992 – 2070)		(2070 – 2100)	
AIS – AGrIS	0.79	710	0.76	1260
GrIS – AGrIS	0.06	-970	-0.46	170

Table S5. Correlations and cumulative differences between the globally averaged C_{ANTH} time series between the combined ice sheet freshwater (AGrIS) and the Antarctic freshwater (AIS) simulations as well as the AGrIS and Greenlandic freshwater (GrIS) simulations calculated for the 1992-2070 and 2070-2100 periods.

	Correlation	Cumulative Difference (Pg C)	Correlation	Cumulative Difference (Pg C)
	(1992 – 2070)		(2070 – 2100)	
AIS – AGrIS	0.97	-11	0.87	24
GrIS – AGrIS	0.89	11	0.99	0.5

Table S6. Normalized root-mean-square error (nRMSE) value ranges and averages across simulations produced by the Gaussian Process Regression (GPR) model between the predicted and actual C_{ANTH} for each predictor variable: Atlantic Meridional Overturning Circulation (AMOC), sea surface salinity (SSS), sea surface temperature (SST), Northern Hemisphere sea ice extent (SIE_{NH}), and Southern Hemisphere sea ice extent (SIE_{SH}).

	OHC_{ANTH}		C_{ANTH}	
Predictor	nRMSE range	nRMSE avg.	nRMSE range	nRMSE avg.
AMOC	0.15 - 0.22	0.19	0.16 - 0.19	0.17
SSS	0.23 - 0.30	0.26	0.23 - 0.33	0.27
SST	0.19 - 0.23	0.20	0.18 - 0.22	0.20
SIE_{NH}	0.16 - 0.22	0.18	0.16 - 0.21	0.18
SIE_{SH}	0.16 - 0.18	0.17	0.15 - 0.20	0.18
